# Green Roof Substrates for Water Quality Improvement: A Critical Review of Biosorption–Phytoremediation Synergies

**DOI:** 10.3390/molecules31111862

**Published:** 2026-05-28

**Authors:** Jordana Georgin, Dison S. P. Franco, Youssef Miyah, Noureddine El Messaoudi, Ashraf M. Al-Msiedeen, Salah Knani

**Affiliations:** 1 Department of Civil and Environmental, Universidad de La Costa, CUC, Calle 58 # 55–66, Barranquilla 080002, Colombia; dstracke@cuc.co; 2 Laboratory of Materials, Processes, Catalysis, and Environment, Higher School of Technology, Sidi Mohamed Ben Abdellah University, Fez 30000, Morocco; 3 Ministry of Health and Social Protection, Higher Institute of Nursing Professions and Health Techniques, Fez 30000, Morocco; 4 Department of Chemistry, College of Science, Imam Mohammad Ibn Saud Islamic University (IMSIU), Riyadh 11623, Saudi Arabia; 5 Center for Scientific Research and Entrepreneurship, Northern Border University, Arar 73213, Saudi Arabia

**Keywords:** green roofs, substrate layer, biosorption, runoff quality, sustainable design, phytoremediation

## Abstract

Green roofs offer significant potential for urban stormwater management, yet their capacity to improve runoff water quality is constrained by the limited pollutant retention of conventional substrates and inherent nutrient leaching risks. This critical review synthesizes recent advances in substrate engineering and phytoremediation to establish an integrated framework for transforming green roofs into active bio-filtration systems. Our analysis reveals that amending conventional substrates with waste-derived biosorbents substantially enhances heavy metal and nutrient retention through complementary mechanisms of surface complexation, ion exchange, and microprecipitation. When strategically coupled with hyperaccumulator plant species and rhizospheric microbial communities, these amended substrates significantly reduce contaminant loads in urban runoff while maintaining hydraulic functionality. We critically evaluate standard growing media versus substrates amended with targeted biosorbents: biochar, which enhances heavy metal retention and hydraulic conductivity via surface complexation; seaweed biomass, which provides superior water retention and cation exchange while reducing synthetic fertilizer dependence; and chitin-rich crab shell waste, which promotes microprecipitation of metals and phosphates while valorizing marine waste. The novelty resides not in the materials themselves, but in their synergistic combination and the systematic comparative analysis of their retention mechanisms under green roof hydrological conditions. This review further identifies critical engineering trade-offs, including biosorbent-induced hydraulic conductivity reductions and long-term adsorption site saturation, and provides actionable design thresholds for amendment dosing, substrate depth, and species selection. Ultimately, this work establishes a mechanistic and practical roadmap for next-generation green roofs that simultaneously optimize stormwater retention, runoff quality, and circular economy valorization, highlighting priority research directions for long-term field validation and climate-adaptive standardization.

## 1. Introduction

Through the use of defined engineering methods, vegetation is planted on the roof of a building; this process is called vegetative roofing. This technology is currently used and aims to restore an ecosystem that has been damaged by uncontrolled civil construction in large urban centers [1,2]. One of the benefits of vegetative roofs or green roofs is to increase the sustainability of buildings through low energy consumption [3,4,5,6]. Other benefits also relate to the surrounding environment and include reducing the maximum flow of water runoff during periods of high precipitation [7,8], the attenuation of heat islands [9], the ecological preservation of cities [10], the weakening of rainwater [11,12], the enhancement of building esthetics [13], the reduction in the building’s energy needs [14,15], improving air quality [16], noise reduction in parallel with acoustic insulation [17], and finally, the protection of the building’s roof membrane [18]. Several developed and even developing countries have begun to use green roofs in large-scale projects in urban metropolises. In the case of France, since 2015, new buildings located in commercial areas must contain photovoltaic panels or green roofs. This policy has already been adopted by countries such as Switzerland, Germany, Japan, Canada, and Denmark. Asian countries (China, Singapore, and Hong Kong) and the United States (USA) have also popularized green roofs. However, their benefits and importance are still not clear and recognized by most countries, as well as by their respective policymakers. One of the reasons may be the lack of local research or very little or nonexistent global research.

An important aspect of these green roofs is the fraction that involves the substrate since it guarantees longevity and stability for sustainable cultivation [19]. In the case of industrial applications, the use of commercial substrates is widespread; the difference with non-commercial substrates is that the latter can be customized with strategic ingredients of preference [19,20]. The proper survival of plants depends heavily on the choice of substrate. For example, if the substrate has a low organic matter content, it will be necessary to add supplements (fertilizers) and water to optimize the growth of the species [21,22]. The construction industry is generally more interested in the economic benefits of increasing customer accommodations, with less interest in sustainability. The guidelines of the German Landscape Research, Development, and Construction Society and the American Society for Testing Materials are generally used for laboratory-scale or greenhouse investigations. In European countries, the most widely used guidelines are the German Landscape Research, Development and Construction Society, which presents limits for chemical and physical properties to increase the efficiency of the ideal output of substrates present in green roofs [23]. Most of these limits were established from pilot-scale experiments carried out in Germany, and therefore are not always fully applicable to other countries in the world, mainly due to climatic differences such as temperature and precipitation [24]. Due to this, the extreme need to establish more modern guidelines to select substrates appropriately was highlighted.

**Figure 1:**
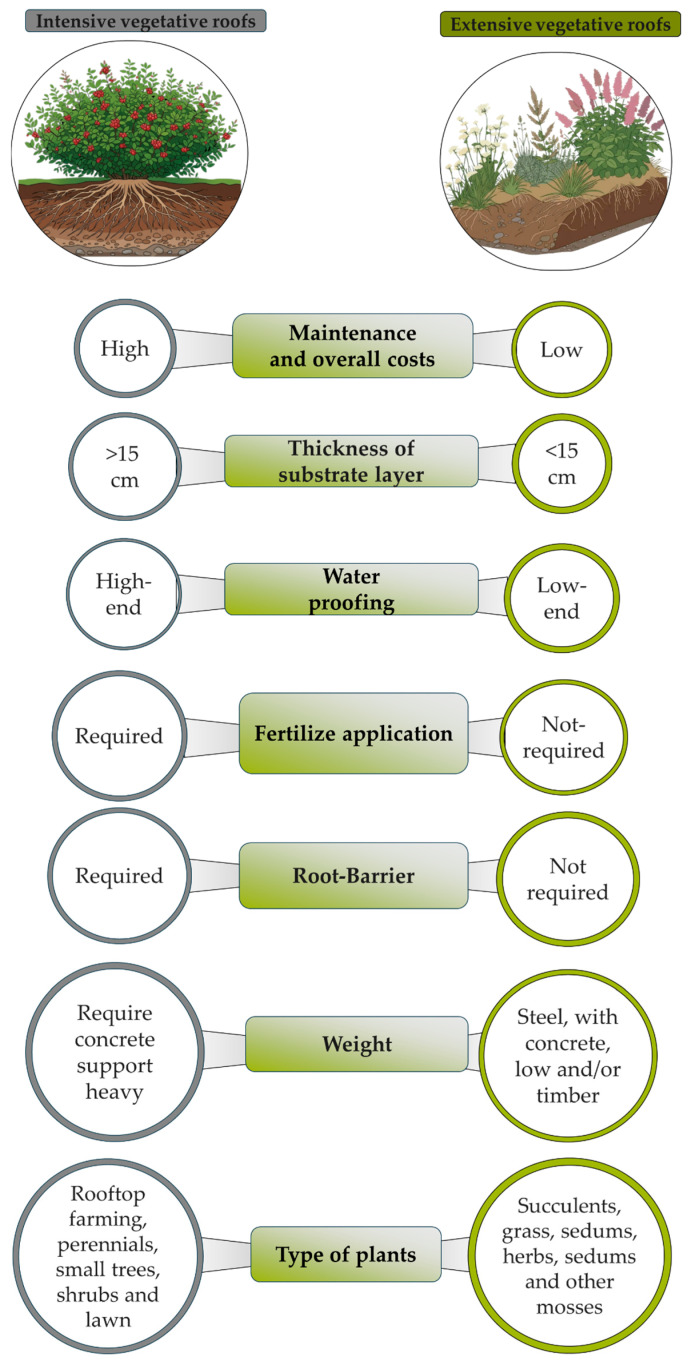
The characteristics of the different types of vegetation cover. Note: The 15 cm substrate depth is a commonly cited threshold distinguishing extensive (<15 cm) from intensive (≥15 cm) systems. In parallel, structural load capacity provides a complementary criterion: extensive roofs typically sustain saturated loads of 60–150 kg m^−2^, while intensive roofs generally require structural support for 180–500+ kg m^−2^, depending on substrate composition, vegetation selection, and intended use (adapted from [28,29]).

Climate comparisons and their limitations are further highlighted in the American Society for Testing Materials guidelines established in 2014 [24,25]. Despite this, the complexity of test analyses in terms of substrate characterization has meant that this standard has been little used by industries. Australia uses AS 4419-2003, as it presents a set of criteria that seeks to evaluate the performance of organic matter, soils, and the inorganic part. Despite this, it is preferable to use AS 3734-2003 in conjunction with AS 4419-2003, seeking to increase the selection of substrates applied to green roofs [26,27]. There is a need to compile all the information that is necessary to establish an adequate growing medium, mainly due to the greater interest in applying green roofs in urban centers. Substrates can be implemented on intensive or extensive roofs. In the case of the latter, such roofs are characterized by a thin layer of substrate, while intensive roofs have a thicker layer to accommodate a greater variety of plant species. Figure 1 briefly illustrates the characteristics of each green roof, with extensive roofs being the most popular and widely distributed throughout the world. While substrate depth is a primary visual and functional differentiator, structural load capacity represents an equally important engineering threshold. Extensive green roofs, designed for minimal maintenance and lightweight construction, typically operate within a saturated load range of 60–150 kg m^−2^. In contrast, intensive systems which accommodate deeper substrates, diverse plant palettes, and recreational use generally require structural support for saturated loads exceeding 180 kg m^−2^, often reaching 300–500 kg m^−2^ in complex installations [23]. These dual criteria (depth and load) should be jointly considered during the preliminary design phase to ensure compatibility with existing building structures.

Although studies highlight the scope of the benefits of green roofs [7,16,30], many have a limited focus on plant growth, which reduces research with analyses of the development of substrates with low weight and subsequent low maintenance (irrigation and fertilization) [31,32]. Because of this, many green roofs suppress all the benefits attributed to them. One solution to this would be to increase the number of investigations that present cooperation between commercial developers and academic researchers. Potential restrictions also interfere with the positive popularization of green roofs. In this sense, the quality of the runoff is evidenced in many studies where several pollutants were detected [32,33,34]. The concentration of contaminants is related to the type of substrate used and the plant species employed [32,35]. Despite this, the number of studies that analyze these elements to improve the quality of runoff water is still insufficient [36,37,38]. While individual amendments such as biochar have been previously explored in green roof substrates, the present review focuses on the strategic integration of three complementary waste-derived materials: biochar, seaweed biomass, and crab shell waste. This selection is driven by their mechanistic complementarity and multi-functional roles. Biochar provides high porosity and surface complexation capacity, seaweed offers exceptional water retention and natural biostimulant properties, and crab shell introduces a distinct microprecipitation pathway via CaCO_3_ dissolution and chitin-mediated binding. The novelty of this work therefore lies in the systematic comparative analysis of their adsorption mechanisms and in proposing a synergistic substrate design that couples biosorption with phytoremediation to simultaneously mitigate heavy metal mobility, nutrient leaching, and hydraulic limitations. Therefore, one of the objectives of this study is to systematically analyze the quality of water runoff from green roofs and describe new techniques for improving this runoff. Another aspect involves the appropriate selection of the substrate for green roofs, which is one of the most important metrics due to the high probability of intense evaporation as a result of the sunlight to which large skyscrapers are subjected daily [39,40]. The chemical and physical properties of the growing media are analyzed, and the existing relationships regarding drought resistance and water tension are also addressed. The primary objective of this critical review is to evaluate how strategic substrate engineering, specifically through the integration of waste-derived biosorbents and hyperaccumulator vegetation, can transform green roofs from passive stormwater managers into active bio-filtration systems for urban runoff quality improvement. To achieve this, the review addresses the following specific research questions: How do conventional green roof substrates compare to biosorbent-amended media in terms of heavy metal retention, nutrient leaching mitigation, and hydraulic performance under realistic runoff conditions? What are the dominant adsorption mechanisms and functional complementarity of key waste-derived biosorbents (biochar, seaweed biomass, and crab shell waste) when applied to green roof substrates? How can the synergistic coupling of substrate biosorption with plant-mediated phytoextraction and rhizospheric microbial activity be optimized to achieve long-term, sustainable runoff remediation? By systematically addressing these questions, this review provides a mechanistic framework and practical design guidelines for next-generation green roofs that align circular economy principles with urban water quality management.

## 2. Literature Search and Selection Methodology

To ensure transparency, reproducibility, and methodological rigor, a systematic literature search was conducted in alignment with the PRISMA (Preferred Reporting Items for Systematic Reviews and Meta-Analyses) guidelines. The primary database search was performed on the Web of Science (Core Collection). The publication window spanned from January 2000 to January 2026, capturing the emergence and technological evolution of green roof substrate engineering, biosorbent amendments, and plant-mediated runoff remediation. The search strategy employed Boolean operators and field tags (e.g., TS = for Topic) to combine keywords across three thematic clusters: (i) Infrastructure: “green roof*” OR “vegetative roof*” OR “living roof*” OR “extensive roof*” OR “eco-roof*”; (ii) Substrate & Remediation Mechanisms: “substrate*” OR “growing media” OR “biochar” OR “seaweed” OR “crab shell*” OR “biosorbent*” OR “phytoremediation” OR “hyperaccumulat*”; (iii) Water Quality: “runoff” OR “stormwater” OR “heavy metal*” OR “nutrient*” OR “water quality” OR “leach*” OR “biosorption”.

**Figure 2:**
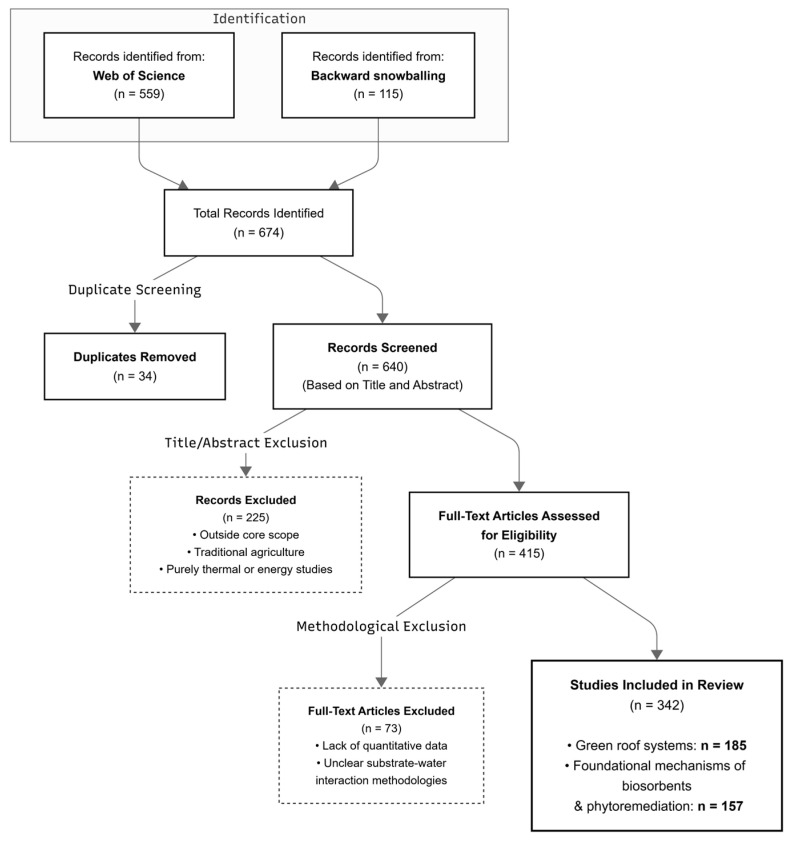
A PRISMA 2020 flow diagram illustrating the systematic literature search, screening, and selection process.

Studies were selected based on the following inclusion criteria: (i) peer-reviewed journal articles or comprehensive reviews; (ii) published in English; (iii) explicitly addressing green roof substrate composition, biosorbent amendments, or plant-mediated runoff remediation; and (iv) reporting quantitative or mechanistic data on pollutant retention, hydraulic performance, or phytoremediation efficiency. Exclusion criteria comprised (i) conference abstracts, editorials, and non-peer-reviewed reports; (ii) studies focusing solely on thermal performance, energy savings, or architectural design without water quality or substrate analysis; and (iii) articles lacking clear methodological descriptions. Given the highly interdisciplinary nature of this review—bridging civil engineering, environmental chemistry, and plant biology, a backward snowballing technique (reference tracking) was systematically employed alongside the database search. This ensured the inclusion of foundational mechanistic studies on biosorbent kinetics and plant-metal interactions that are not always indexed under “green roof” keywords but are critical for the proposed synergistic framework. This structured, hybrid approach minimizes selection bias, ensures comprehensive coverage of both applied engineering and fundamental mechanisms, and strengthens the validity of the proposed framework. The complete selection process is illustrated in the PRISMA flow diagram (Figure 2).

## 3. Components Present in Green Roofs

**Figure 3:**
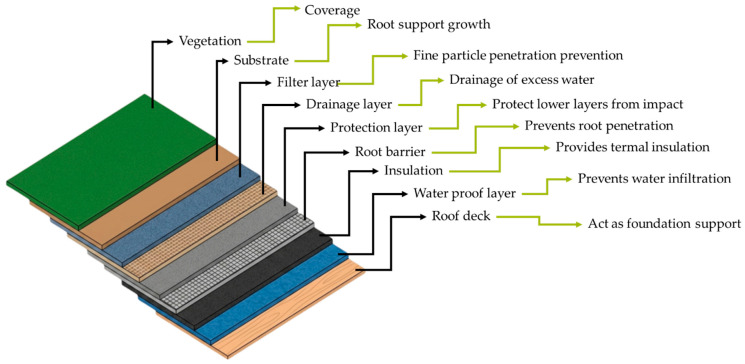
An illustration of a general diagram of the components present in a green roof.

A green roof engineering project involves several components, including the plant species present on top, the type of growth substrate presents just below, the filter followed by the drainage element, and, finally, the membrane (waterproof). Some projects also have a protective layer, an insulation layer, or even a protective barrier for the root system (present in intensive roofs). Figure 3 represents a green roof system diagram; here, it is worth noting that the success of the system, where sustainability issues are involved, will depend heavily on the combination of all these factors [34,41]. Each component of a green roof has specific selection criteria that ensure proper system performance. The plant species form the uppermost layer and strongly influence the visual appearance, thermal performance, mitigation of urban heat islands, and air quality benefits of the green roof system [42,43]. Guidelines for plant selection are essential to ensure adaptation to harsh roof conditions, such as arid climates and extreme temperatures. The substrate also plays a key role by supporting plant growth, improving rainwater retention and runoff reduction, while contributing to acoustic insulation and energy savings.

**Figure 4:**
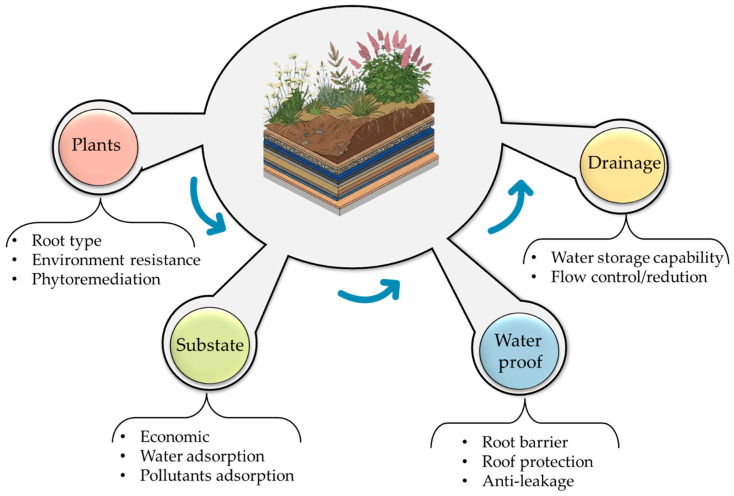
The main components present in a green roof and their particularities.

As shown in Figure 4, a variety of organic and inorganic components are mixed in varying proportions to generate a substrate with desirable properties [44]. The drainage layer prevents waterlogging and creates favorable conditions for plant growth by maintaining proper aeration. During drought periods, stored water in drainage materials can support vegetation, while the system also improves thermal performance and protects the waterproof membrane [4,21]. The filter layer separates the substrate from the drainage system, preventing clogging caused by substrate particles. Its presence also enhances the overall water retention capacity of green roofs [45,46]. Green roofs require waterproofing due to constant moisture. Intensive systems often include a root barrier, and an optional insulation layer can improve thermal regulation. Table 1 summarizes the main components and their characteristics.

Runoff water quality from green roofs can vary according to the substrate and plant species used, and studies have reported the presence of various pollutants in this runoff [32,34,61]. Because rainwater is naturally relatively clean, containing only trace metals, nutrients, and slightly acidic pH, any pollution introduced by green roof runoff becomes more significant and concerning. Researchers frequently report that green roofs generate runoff with high concentrations of metals [60], high Total Dissolved Solids (TDSs) [62], high nutrient discharges [63], and Total Organic Carbon (TOC) [64,65]. A study in China reported that green roof runoff increased levels of organic matter, nutrients, and trace metals, including TOC, COD, BOD, nitrogen species, major ions, and metals such as lead and manganese [65]. As a positive aspect, the study reported neutralization of rainwater pH and reduced TSSs, while other studies in Sweden also identified green roofs as sources of significant heavy metal discharge [53,66].

It is important to clarify that heavy metals such as lead, manganese, copper, zinc, and cadmium detected in green roof runoff are not produced by the vegetation itself, but originate from external anthropogenic and environmental sources. The primary pathways include (1) atmospheric deposition (urban air pollution from vehicle exhaust, industrial emissions, brake and tire wear, and fossil fuel combustion releases metal-laden particulate matter that settles onto roof surfaces via dry deposition or is scavenged by precipitation); (2) precipitation inputs (rainwater, while relatively pure, can contain trace concentrations of metals derived from atmospheric aerosols, especially in industrialized or high-traffic urban areas); (3) substrate and amendment leaching (certain inorganic aggregates (e.g., crushed brick, expanded shale) or organic amendments (e.g., composts, biosolids) may contain background levels of metals that can be mobilized under specific pH or redox conditions); (4) roofing infrastructure (corrosion of metal flashings, fasteners, drainage components, or waterproofing membranes can contribute metals such as zinc, copper, or lead to runoff, particularly during initial weathering phases); and (5) fertilizers and maintenance inputs (synthetic or organic fertilizers, pesticides, and irrigation water may introduce trace metals, especially if derived from recycled or industrial by-products).

Understanding these external sources is critical for interpreting runoff quality data and for designing green roofs that function as effective bio-filtration systems. The biosorbent amendments and phytoremediating species evaluated in this review are intended to intercept, retain, or transform these externally sourced contaminants, thereby improving the quality of discharged runoff relative to conventional roof systems. Table 2 summarizes runoff water quality from green roofs in different regions. Water quality is a critical aspect that must be carefully considered in green roof design, as neglecting it may compromise project sustainability and contribute to a negative perception of these systems.

**Figure 5:**
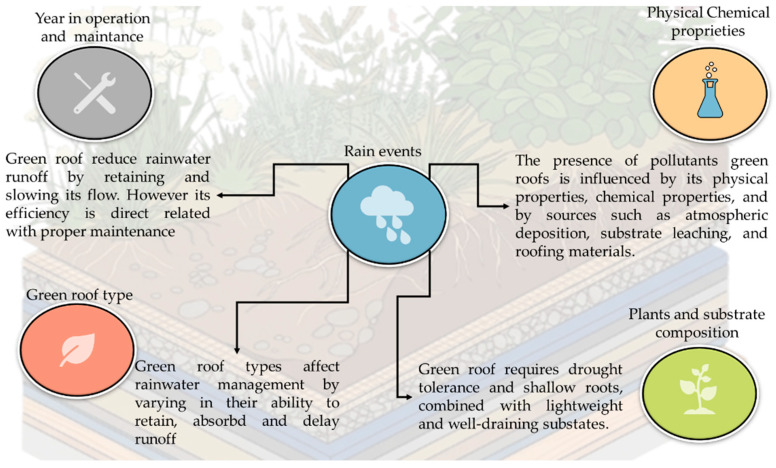
The influence of different factors on the quality of water runoff on green roofs.

Green roofs can reduce rainwater quality through processes such as water percolation through vegetation and substrate, where it may become either filtered or contaminated [68,69]. Plants can improve rainwater quality by capturing dust and airborne particles, helping to purify precipitation. The substrate also acts as a filter, promoting ion exchange that can remove or release metals, nutrients, and other pollutants present in rainwater [70]. Roof components can act as sinks, reducing ion concentrations in runoff water [71]. When ion concentrations in rainwater are lower than those in the green roof substrate, ions may be released into the runoff, increasing its concentration [72]. As a result, roof runoff may contain higher ion concentrations than incoming rainwater. Soil microorganisms and fertilizer inputs can also contribute to increased ion levels in the runoff [73]. Fertilizers can leach into runoff depending on plant uptake and release mechanisms, while microbial activity also significantly affects water quality. Figure 5 illustrates the main factors influencing runoff quality, with substrate composition and plant species being the most critical [74]. Both factors must be carefully managed to achieve the desired runoff water quality [75]. Although important, few studies focus on strategies to improve runoff quality. Therefore, this review emphasizes the roles of substrate and plant components in detail. The following subsections systematically examine substrate proportions, plant selection criteria, growth assessment methodologies, and key physicochemical properties. This descriptive foundation precedes a targeted critical synthesis (Section 3.5), where empirical evidence from Section 3.1, Section 3.2, Section 3.3 and Section 3.4 is integrated to identify methodological limitations, knowledge gaps, and priorities for future substrate engineering.

### 3.1. The Proportion of the Mixture Present in the Substrate

This section reviews commonly used growing media and their proportions in previous studies. Proper formulation improves plant nutrition and water infiltration, while preventing excessive weight that could compromise building durability [76,77,78]. Table 3 describes the details of the mixture compositions and their proportions, which were used to design substrates used in previous investigations.

Regional context is important in green roof design, as climate, plant species, and roof type (intensive or extensive) influence the optimal substrate composition and overall performance, including weight and the benefits achieved [19,80]. Prototypes can be used to optimize substrate mixtures before installation, and substrate thickness is also crucial for green roof durability. The substrate depths used in the following studies were determined based on the water retention capacity, as being 8, 10, 14, 15, 16, and 20 cm in the study by Soulis et al. [79], <15 cm for the study by Rowe et al. [81] 2.5, 5 and 7.5 cm used in the study by Kader et al. [27], and finally, depths of 2.5, 4 and 6 cm were used in the study by VanWoert et al. [82]. Comparative studies show that a 4 cm substrate depth provides higher water retention, although its thermal performance was not evaluated [81,83].

### 3.2. Plant Selection

Plant selection should consider local climate and resource availability. Studies often choose species adapted to regional conditions; for example, *Dianella admixta*, *Stypandra glauca*, and *Lomandra longifolia* were selected in green roof experiments due to their high tolerance to thermal radiation [84]. In smaller numbers, it is also possible to observe studies that used seeds sown on green roofs [85,86] and seeds added together with plant residues, as a form of fertilizer [87]. In some cases, plant selection is guided mainly by local climate and availability, with minimal restrictions. For example, *Bouteloua dactyloides* (buffalo grass) is widely used in Sri Lanka due to its adaptability to local conditions. It is favored for green roofs because it is low-maintenance, disease-resistant, and requires little water [27]. Due to its characteristics, *Bouteloua dactyloides* is recommended for preliminary studies on substrate composition. Plant development has been assessed through parameters such as leaf abscission, survival rate, and growth rate, often using aerial imagery [88]. For more robust analysis, plant growth should be evaluated using variables such as height, stem diameter, flower proportion, and the number of blooms [57,89]. Since some studies on *Bouteloua dactyloides* did not consider growth and survival parameters, plant height is recommended as a key metric in preliminary evaluations, measured from ground level to the plant apex across different substrates.

Drought resistance has been assessed across species using different methods, including binary classification, where plants are scored as 0 (dead) or 1 (alive), typically based on long-term visual observation [90]. The experiment ends by calculating plant survival rates, though this method is limited for short-term drought studies and may involve observational errors. Visual assessment has also been used in controlled green roof experiments with multiple species over extended periods [91]. During the first 90 days, plant growth was monitored under controlled irrigation, followed by natural development without parameter control. Growth was assessed only through photographic visual analysis, which may reduce reliability due to its subjective nature and lack of quantitative measurement [92]. All studied species allow for individual separation, unlike buffalo grass (*Bouteloua dactyloides*), which has a dense, interconnected root system. As a result, standard individual-based assessment methods are not suitable for this species [93]. A more suitable approach is to assess survival based on vegetation cover area rather than counting individual plants.

### 3.3. Methodologies for Determining the Growth of Plants Present on Green Roofs

Plant growth is assessed using varied methods, including long-term measurements and visual estimates of coverage across different species [94]. In another study, weekly photographic monitoring during the growing season was used to assess survival, leaf loss under drought, and growth during wet periods [88]. This method is suitable only for species with horizontal growth, such as buffalo grass, and not for vertical growth measurement. Therefore, aerial imagery is not appropriate for assessing growth rates in this study. Another study proposed an alternative method to evaluate vegetation growth [90]. A 3D pin method was used to estimate vegetation cover by comparing initial and final pin contact counts, but it is not broadly applicable to other species due to methodological differences. Other studies assess growth using traits such as plant diameter, height, and flower production [95,96]. In another study, growth was assessed solely by measuring plant height from the base to the highest leaf apex [86]. Overall, standardized methods are needed to improve reliability and reduce errors. However, given the structural diversity of plant species used in green roofs, multiple complementary methodologies are necessary to account for morphological and genetic differences across species in both intensive and extensive systems [97]. Based on the comparative analysis in Table 4, we recommend selecting growth assessment methodologies according to three key criteria: (i) plant morphology ground-cover species (e.g., Sedum, buffalo grass) are best evaluated via vegetation cover area or UAV-NDVI, while discrete individuals (e.g., grasses, forbs) benefit from morphometric measurements; (ii) research-scale pilot studies can employ pin structures or manual measurements, whereas large-scale or long-term monitoring favors photographic analysis or UAV-based approaches; and (iii) target metric survival studies may use binary classification, while studies linking growth to ecosystem services (e.g., runoff retention, thermal performance) should prioritize quantitative, continuous metrics such as coverage percentage or biomass proxies. For studies aiming to standardize comparisons across species or sites, we advocate adopting a hybrid approach: combining simple height measurements (for rapid screening) with periodic photographic coverage analysis (for canopy-level validation), calibrated against a subset of harvested biomass samples where feasible.

### 3.4. Important Properties of an Ideal Substrate

#### 3.4.1. Rainwater Retention

Studies using similar models and varying dimensions have evaluated rainwater retention in green roof substrates. Platforms measuring 0.67 m × 2.44 m were used to analyze the effects of substrate surface, retention layer thickness, and slope [82]. Platforms measuring 1 m × 2 m were used to evaluate the effectiveness of green roofs in reducing surface runoff [79]. Another study included moisture content and temperature as additional parameters to assess their influence on rainfall water retention [99]. The three studies used a 2% platform slope, representing typical substrate conditions. Precipitation and runoff were measured using different methods, including a prototype designed to simulate combined sewer overflow and stormwater reduction scenarios [100]. Rainfall and runoff monitoring in green roof studies commonly uses specialized instruments, including tipping bucket rain gauges, soil moisture sensors, drainage channels, and ultrasonic water level sensors. While accurate, some equipment is costly and less suitable for low-budget applications. In other studies, runoff collection was simplified using greenhouse setups with controlled indoor–outdoor temperature gradients [57]. The study used simulated water input, eliminating the need for separate precipitation measurement, unlike natural rainfall experiments where this step is required. In a greenhouse setup, temperature differences were maintained between indoor (>293 K) and outdoor conditions (<293 K). Another study showed that aluminum foil gutters installed at platform outlets effectively guided runoff collection [82]. Aluminum foils were segmented according to the experimental design, and runoff was measured using tipping bucket rain gauges. Larger-budget studies often relied on weather stations for rainfall data, although accuracy can be affected when stations are located far from the experimental site [94].

Synthesizing the experimental evidence reviewed above, this section proposes a set of ideal design parameters for optimizing rainwater retention in green roof substrates. Based on cross-study comparisons and alignment with established guidelines (FLL 2002; ASTM E2777-14), the following parameters are recommended for extensive green roof systems: (1) (substrate depth) For maximum retention without compromising structural load, a depth of 8–12 cm is optimal for extensive roofs in temperate climates, balancing water storage capacity (~25–35% vol.) with weight constraints (<150 kg m^−2^ saturated). In arid or semi-arid regions, depths of 12–15 cm are preferable to enhance drought resilience, provided structural support permits. (2) (slope and hydraulic conductivity) A roof slope of 1.5–3% is ideal to prevent ponding while maintaining sufficient contact time for infiltration. Saturated hydraulic conductivity should exceed 3600 mm h^−1^ (FLL minimum) but remain below 10,000 mm h^−1^ to avoid rapid drainage that reduces retention efficiency. (3) (water retention capacity and porosity) Target a volumetric water retention capacity of ≥30% at field capacity, with air-filled porosity maintained at 15–25% to ensure root aeration. Substrates amended with biochar (5–10% *v*/*v*) or seaweed biomass (≤10% *v*/*v*) can achieve these targets while enhancing pollutant sorption. (4) (initial moisture content and vegetation effects) Retention performance is maximized when substrates are at 40–60% of field capacity prior to rainfall events. Dense vegetation cover (>80% canopy closure) further enhances retention via interception and evapotranspiration, reducing runoff volume by an additional 10–20% compared to bare substrates. These parameters should be adapted to local climatic conditions: in high-precipitation regions, prioritize hydraulic conductivity to prevent saturation; in drought-prone areas, emphasize water retention and drought-tolerant vegetation. Future pilot-scale studies should validate these thresholds under dynamic, multi-season conditions to refine climate-specific design protocols.

#### 3.4.2. Substrate Density

Apparent density can be measured using direct and indirect methods. Direct methods include excavation, nuclear, and core techniques, while indirect methods involve regression and radiation approaches [101]. A study compared direct methods for measuring apparent density across different soils used for crop growth, finding them suitable overall, with the core method being best for undisturbed soils [27]. Indirect methods are less commonly used due to high cost, although substrate disturbance in cultivation media often limits direct methods and favors indirect approaches. For sampling, structured sampling is most appropriate, as it is widely used to assess soil compaction in relation to apparent density [102]. A 0.5 m^2^ stainless steel frame was inserted into the soil to define a reference plane, after which a known soil thickness was manually removed and surface elevation was re-measured. These measurements allowed for the calculation of removed soil volume, and combined with mass data, enabled determination of soil density [27]. Density can be measured by different methods depending on the study, and substrate selection should prioritize low weight and suitable moisture properties [56,103,104]. The apparent density values discussed herein directly inform the saturated load calculations referenced in Figure 1. For extensive systems targeting the 60–150 kg m^−2^ range, substrate formulations must prioritize lightweight inorganic aggregates (e.g., expanded shale, pumice) with dry densities <1200 kg m^−3^ and wet densities <1800 kg m^−3^, consistent with FLL guidelines [23].

#### 3.4.3. Thermal Performance

The literature includes studies assessing the thermal performance of green roofs, including comparisons between buildings with and without green roof systems [105,106]. Temperatures were measured using infrared thermometers and air temperature was estimated with a psychrometer, showing that green roofs reduce temperature fluctuations and improve thermal performance, helping to lower energy consumption. Another study compared grass-covered and bare concrete slabs to evaluate thermal effects [27]. Green roofs improved thermal performance in experiments comparing covered and uncovered slabs, with measurements of internal, surface, and intrados temperatures under sunny conditions. Temperature gradients within the substrate were also assessed using thermistors placed at multiple depths, depending on roof thickness [84]. Another study used T-type thermocouples and DS18B20 sensors to measure temperatures beneath the green roof and at the surface [107]. Substrate bottom and surface temperatures were continuously recorded, demonstrating high measurement accuracy.

While the studies cited above demonstrate the thermal benefits of green roofs in general, it is important to distinguish how thermal performance differs between intensive and extensive systems. Intensive green roofs, characterized by deeper substrates (>15 cm), diverse vegetation (including shrubs and small trees), and higher biomass, typically exhibit superior thermal insulation and heat capacity compared to extensive roofs. The greater substrate depth increases thermal mass, dampening diurnal temperature fluctuations and reducing heat transfer to the building interior by up to 30–40% relative to extensive systems under comparable climatic conditions [105,106]. Additionally, the complex canopy structure of intensive roofs enhances shading and evapotranspiration, further lowering surface temperatures by 5–10 °C during peak solar radiation [108].

In contrast, extensive green roofs with shallow substrates (6–15 cm), low-growing vegetation (e.g., *Sedum* spp., grasses), and minimal maintenance offer more modest but still significant thermal benefits. Their lighter weight and lower cost make them suitable for retrofits, where even a 2–4 °C reduction in roof surface temperature can translate to measurable energy savings in cooling-dominated climates [109]. However, their limited substrate depth restricts water storage capacity, reducing evapotranspiration-driven cooling during prolonged dry periods. Consequently, the thermal performance of extensive roofs is more sensitive to irrigation frequency and climatic variability. From a design perspective, the choice between intensive and extensive systems should align with thermal performance goals and structural constraints. In hot, arid climates, intensive roofs with irrigation support maximize cooling via evapotranspiration; in temperate or cold climates, extensive roofs with high-albedo substrates may suffice for reducing winter heat loss. Future studies should quantify the long-term thermal degradation of substrate amendments (e.g., biochar, seaweed) under cyclic wet–dry conditions to refine climate-specific design protocols for both roof types.

#### 3.4.4. Thermal Conductivity

Thermal properties are widely used in agriculture, materials science, and engineering, with recent studies increasingly focusing on determining the thermal conductivity of soil aggregates [110,111]. Soil thermal properties influence microclimate and plant development processes such as establishment, emergence, and germination. Thermal conductivity depends on both intrinsic soil characteristics and external factors, with moisture content being the most difficult to control [112,113,114,115]. Since the 1980s, studies have increasingly focused on the influence of moisture on soil thermal conductivity [116,117]. Studies indicate that thermal conductivity is higher under high moisture conditions, as water facilitates heat transfer between mineral particles. This behavior is also observed in saturated sediments like loess, where liquid bridges between particles enhance conductivity compared to dry soils [118]. Higher moisture increases soil thermal conductivity, while higher salt concentrations can reduce it [118,119,120,121,122]. A quartz sand sample showed higher thermal conductivity at greater moisture content compared to a lower-humidity sample [123]. Substrate wetting agents significantly influence the thermal conductivity of substrates.

The method for measuring soil thermal conductivity based on temperature change and decay was originally developed in soil analysis studies [27]. The method was later improved by incorporating volumetric heat capacity and double-probe techniques, accounting for aggregate morphology and volumetric proportions [120,124]. The double probe process uses two-needle probes positioned in parallel [125]. The double-probe method uses one probe as a heater and another as a temperature sensor. Following ASTM D5334, it has been applied to measure green roof thermal conductivity under both wet and dry conditions [126]. Wet soils have higher thermal conductivity than dry soils. The needle probe method is accurate but complex and costly, while the Lee disk method is a simpler alternative [127,128,129]. The Lee method is not suitable for culture media because their heterogeneous components cannot be molded into uniform disk-shaped specimens [128]. Even if molding was possible, the heterogeneity of substrate aggregates would still cause significant variability in thermal conductivity measurements using this method [128,130].

#### 3.4.5. pH Value Present in Substrates

Substrate pH is important because values outside optimal crop ranges can cause nutrient deficiencies and plant damage, making pH control essential for crop health [131]. pH in soils is commonly assessed using standardized methods (e.g., ASTM E70), and substrate pH values should be compared with ambient conditions and vegetation requirements reported in the literature to evaluate green roof feasibility. Studies also show that pH influences nutrient availability, with optimal ranges varying by crop type [132]. It is important to distinguish between generalized substrate guidelines and species-specific pH requirements. While the FLL recommends a pH range of 6.0–8.5 to ensure broad compatibility with extensive green roof vegetation and minimize risks of nutrient lockout or metal mobilization, certain ornamental species exhibit optimal performance at lower pH values. For instance, acid-preferring taxa such as Azalea (pH 4.5–5.8) and Blue hydrangea (pH 5.2–5.6) are included in Table 5 to illustrate how substrate chemistry can be strategically manipulated to achieve targeted esthetic outcomes (e.g., flower coloration via aluminum bioavailability). In green roof design, substrate pH should generally align with the conservative FLL range unless the project explicitly incorporates acid-tolerant ornamentals with corresponding maintenance protocols to sustain long-term system functionality. New growing media should be evaluated for pH through comparative studies with similar substrates to better assess their suitability for green roof applications [133]. Due to limited studies on coconut fiber substrates, pH comparisons can be made with more studied materials such as wood sawdust, since both are plant-based residues with potentially similar properties. The species listed in Table 4 were intentionally selected to illustrate the critical relationship between substrate pH and plant performance. These crops represent commonly cultivated greenhouse species with well-documented pH requirements, spanning acid-preferring taxa (e.g., Azalea, pH 4.5–5.8) to neutral-adapted species (e.g., Geranium, Marigold, pH 6.0–6.8). Importantly, several entries (e.g., *Hydrangea* spp.) demonstrate pH-dependent ornamental traits, underscoring how substrate chemistry directly influences esthetic outcomes a consideration increasingly relevant to green roof design in urban landscaping. While derived from greenhouse literature, these pH thresholds provide a transferable framework for evaluating substrate suitability for green roof vegetation, particularly given the shared physiological constraints (shallow root systems, drought tolerance, low nutrient demands) between greenhouse ornamentals and extensive green roof species.

Phosphoryl, nitrile, and carbonyl groups formed through interactions between organic matter and minerals influence substrate chemistry by reducing electronegativity and affecting hydrogen dynamics in pore water, which can increase pH. According to construction guidelines, the optimal pH for plant growth ranges from 6 to 8.5 [134,135]. These pH values are considered appropriate depending on substrate type and depth, particularly in the context of extensive green roof performance [136]. A pH around 7 is generally considered ideal for plant growth, making near-neutral substrates most suitable for green roofs because they avoid both acidic and alkaline stress conditions [137]. This also reduces future soil correction costs, making substrates closest to pH 7 the most suitable in terms of stability and plant growth conditions.

#### 3.4.6. Electrical Conductivity of the Substrate

Salinity in aqueous solutions is commonly estimated through electrical conductivity measurements [138]. In environmental engineering, agriculture, and materials science, salinity in culture media is a key parameter, as high levels can cause cytotoxicity, nutrient imbalance, and water stress due to elevated Na^+^ and Cl^−^ concentrations [139]. High salinity negatively affects soil health and plant growth, reducing its productive potential. Therefore, it is essential to ensure salinity remains below recommended limits. Increased soil salinity, driven by factors such as erosion, industrial pollutants, plastics, and contaminated water, has been shown to impair seed germination and plant development [140,141,142,143,144,145]. Salinity imbalance can cause ionic toxicity, nutrient deficiencies, and osmotic stress, negatively affecting plant growth [146]. Better methods are needed to measure electrical conductivity as an indicator of salinity, which is commonly assessed using soil and substrate composition [147]. Higher concentrations of mineral salts increase electrical conductivity due to the greater presence of ions in solution [148,149]. Drainage water from substrates often shows high salinity, which can be further intensified in dry climates [138,150]. Evaporation concentrates residual salts, increasing conductivity in the drainage water. However, due to limited studies on conductivity in culture media, more comprehensive research is needed for reliable conclusions. Electrical conductivity meters quantify ionic charge in solution, reflecting dissolved minerals and overall salinity in soil or water. A common measurement method is a 1:2 dilution, mixing one part soil or culture medium with two parts distilled water [151]. Tap water cannot replace distilled water due to its mineral ion content. In this study, electrical conductivity was measured using a multimeter in solution, although a limitation is its sensitivity to temperature variations. Another approach for assessing conductivity is the Pour-Through technique [152,153,154,155]. The Pour-Through technique involves saturating the substrate, allowing it to rest for 120 min, and then collecting about 100 mL of leachate by adding distilled water. The water volume depends on the container size and substrate type [156,157]. The Pour-Through technique extracts leachate without disturbing the substrate but is time-consuming and yields conductivity readings affected by external conditions.

Modern electrical conductivity measurement uses direct sensors with signal-processing algorithms to detect conductivity at the soil–electrode interface [155,158,159,160]. Direct sensors are a modern, efficient, and relatively low-cost method for measuring conductivity in laboratory setups. However, battery levels must be monitored to maintain accuracy, and readings are typically averaged over 60 min after irrigation to reduce variability [27]. Even with corrections, sensor-based electrical conductivity values may not match absolute measurements. While direct sensors are effective in small-scale systems, readings can be distorted near fertilizer prills, requiring repeated measurements in different locations. The Saturated Medium Extract method is more commonly used in laboratories to obtain standardized conductivity values [161]. The procedure involves saturating the sample with distilled water, collecting the extract, and analyzing the resulting solution. In practice, field samples are saturated, then squeezed through gauze to obtain the liquid extract for measurement [161]. The Saturated Medium Extract method has limitations, as it does not account for moisture effects on conductivity, uses a fixed water ratio, is time-consuming, and is not suitable for bare-root growing media.

#### 3.4.7. Nutrient Content of Substrates

TDSs represent the overall mineral content of a substrate solution. When TDSs are within an adequate range and pH is optimal, nutrient uptake by roots occurs more efficiently [162]. High pH and elevated dissolved solids can disrupt nutrient uptake in plants [163]. Therefore, substrate selection for green roofs should consider more than just total dissolved solids. In aqueous soil extracts, dissolved solids are commonly assessed using electrical conductivity meters [164]. To find the abundance of phosphate and nitrate, various methods have been adapted, such as voltammetry [165], spectroscopy [166], and chemical sensors [167]. Nitrates are important for plant growth as they promote chlorophyll production and enhance photosynthetic energy generation [168,169]. Nitrates in the substrate contribute to nitrogen nutrition, as plants absorb ammonium and nitrate to synthesize amino acids and proteins. This availability supports photosynthesis and overall plant growth [169]. Phosphorus in culture media supports the development of plant structures such as roots, flowers, and fruits. It is commonly supplied as orthophosphates derived from interactions between organic matter, minerals, and fertilizers in the substrate [170]. Phosphorus availability is strongly influenced by substrate pH. Under alkaline conditions, its uptake is reduced, while near-neutral pH (6.5–7) favors its reactions with aluminum and iron, leading to the formation of compounds such as AlPO_4_ and FePO_4_ [171]. Near-neutral pH combined with adequate phosphate and nitrogen levels enhances plant energy production by supporting efficient photosynthesis under suitable light conditions.

### 3.5. Discussion and Critical Analysis of the Important Properties of an Ideal Substrate

Building on the empirical data presented in Section 3.1, Section 3.2, Section 3.3 and Section 3.4, this subsection synthesizes four critical limitations that currently constrain green roof substrate optimization: (1) a lack of prioritization of parameters and climate specificity, (2) excessive reliance on short-term experiments and controlled conditions, (3) insufficient integration between thermal, structural, and hydraulic performance, and (4) the need for standardized and context-oriented evaluation frameworks. These four gaps underpin the rationale for the integrated approach proposed in this review (biosorption + phytoremediation) and guide recommendations for future research. According to this study, several variables have a significant impact on the selection of appropriate substrate species, namely substrate nutrient content, substrate composition mixing proportions, salinity, substrate layer thickness, pH, green roof vegetation type, thermal conductivity, drought resistance, thermal performance, substrate growth contribution, substrate density, and rainwater retention. The distinction between general guidelines for substrates and specific optimal pH values for each species (Table 4) highlights the gap regarding the lack of parameter prioritization frameworks adapted to specific climatic and design contexts. Future guidelines should differentiate between reference resilience thresholds and performance optimization targets for specialized applications.

The literature does not provide details on which of these parameters requires greater attention because it has the greatest influence. However, synthesizing the empirical evidence and methodological limitations reviewed in Section 3.1, Section 3.2, Section 3.3 and Section 3.4, this review proposes a hierarchical prioritization framework to guide future engineering and substrate standardization efforts. Based on their direct impact on system viability, surface runoff quality, and structural feasibility, the parameters are classified as follows: (1) Hydraulic Performance (Water Retention Capacity and Saturated Hydraulic Conductivity): This has primary control over peak stormwater attenuation, surface runoff pollutant leaching, and prevention of surface ponding or anaerobic microzones. Without adequate hydraulic balance, all other substrate functions are compromised. (2) pH and Electrical Conductivity (Salinity): They are direct determinants of plant viability and nutrient bioavailability. (3) Substrate pH outside the range of 6.0–8.5 and high EC levels induce immediate phytotoxicity, nutrient lockout, or excessive ion leaching, making them non-negotiable basic constraints. (4) Apparent Density and Layer Thickness: These factors determine structural load limits and the feasibility of adapting existing buildings. Lightweight media (<1500 kg m^−3^ wet for extensive systems) are structurally mandatory, restricting the permissible proportions of organic fertilizers. (5) Nutrient Content and Mixing Ratios: These factors govern long-term fertility, microbial activity, and pollutant retention, but must be carefully balanced with hydraulic and structural limits. Excessive organic fractions increase nutrient leaching, while mixtures excessively dominated by minerals compromise water retention and plant establishment. (6) Thermal Conductivity and Performance: They are highly valuable for building energy efficiency and membrane protection, but strongly dependent on moisture and secondary to hydrological and chemical stability. (7) Drought Resistance and Growth Contribution: They are climate-dependent and increasingly controllable through biosorbent fertilizers, irrigation strategies and selection of specific species, rather than just by substrate formulation.

This proposed classification alters the design paradigm, moving from the optimization of isolated parameters to a systems engineering approach, where hydrological stability and chemical compatibility constitute the fundamental constraints. Future guidelines (e.g., FLL, ASTM) should adopt this hierarchical structure to develop performance-based and climate-adaptable substrate standards.

As detailed in Section 3.1, while various mixture proportions have been tested (ranging from 15% attapulgite clay to 65% pumice, and from 40% heat-expanded slate to 40% sand), these studies do not establish which specific proportion optimizes the trade-off between water retention capacity and structural stability across different climatic conditions. It is known that sandy soils have a greater tendency to evapotranspiration; therefore, a substrate with high resistance to drought periods and with a high water retention potential is recommended, which is highly important in the case of green roofs located in regions such as Saudi Arabia [24]. However, the proportional analyses presented in Section 3.1 do not clarify whether a 65% pumice mixture would perform adequately in arid climates compared to a 40% sand mixture in Scandinavian regions. This type of substrate that has a high water retention potential is not necessarily necessary in green roofs located in Scandinavian countries or Iceland. However, in these areas, the substrates must have low salinity and high nutrient content, due to the natural conditions of these regions having soils with poor fertility and the presence of various contaminants generated by human pollution [172,173,174,175]. In this regard, it is recommended that future studies focus on this condition (anthropogenic pollution and low fertility) to enable us to choose green roof substrates that adapt to these limitations.

Most investigations aim to detect the feasibility of using various substrates based on different aspects, such as amendments with substrates such as hydrogels [176], organic matter [177,178,179] and perlite [180], different substrate depths [181,182,183,184], climatic variables (temperature and precipitation) [185,186,187,188,189] and water retention. However, as highlighted in Section 3.3, the methodologies for determining plant growth lack standardization. While some studies measured growth seven times over approximately two years using visual inspection [94], others employed three-dimensional pin structures or simple height measurements from base to apex. This methodological inconsistency, detailed in Section 3.3, prevents direct comparison between studies and obscures which growth assessment protocol provides the most reliable indicator of substrate performance. Most investigations are confined to greenhouse or pilot-scale setups with tightly controlled variables. This limits the understanding of dynamic, season-dependent changes in substrate properties, plant-substrate interactions, and long-term pollutant retention/desorption behavior. The absence of extended field monitoring compromises the extrapolation of laboratory findings to real-world urban installations.

The physicochemical property analyses presented in Section 3.4 reveal critical knowledge gaps regarding long-term performance. While Section 3.4.3 demonstrated that green roofs can reduce thermal fluctuations and increase thermal capacity [105,106], and Section 3.4.4 showed that wet soils present higher thermal conductivity compared to dry fractions [126], these thermal performance studies do not address how pore size distribution interacts with thermal conductivity over multi-year dry–wet cycles. Similarly, while Section 3.4.2 discussed density measurement methods (direct vs. indirect), and Section 3.4.5 established that pH values between 6 and 8.5 are ideal according to FLL guidelines [134,135], the interplay between substrate density, pH stability, and structural load-bearing capacity remains underexplored. Long-term thermal performance analyses, particularly under extreme diurnal temperature fluctuations, are rarely coupled with substrate engineering, leaving a critical gap in predicting energy-saving potential and membrane protection over the roof’s lifespan. The plant selection criteria discussed in Section 3.2, combined with the nutrient content analysis in Section 3.4.7, highlight the need for context-driven evaluation frameworks. While Section 3.2 identified species such as *Dianella admixta*, *Stypandra glauca*, and *Lomandra longifolia* as suitable for thermal radiation resistance [84], and Section 3.4.7 established that phosphorus absorption is strongly dependent on substrate pH (with optimal conditions between 6.5 and 7) [171], current selection practices lack a unified methodology that balances lightweight requirements, hydraulic conductivity, salinity thresholds, and plant compatibility across diverse urban climates. Bridging this gap requires transitioning from controlled-parameter studies to realistic, multi-season evaluations that account for climatic variability, maintenance constraints, and economic feasibility.

Collectively, the descriptive data presented in Section 3.1 through Section 3.4 provide the empirical foundation necessary to identify these four critical limitations. The mixture proportions (Section 3.1), plant selection criteria (Section 3.2), growth assessment methodologies (Section 3.3), and physicochemical properties (Section 3.4) collectively demonstrate that while substantial progress has been made in characterizing green roof substrates, the field lacks (1) parameter prioritization frameworks adapted to specific climatic contexts, (2) standardized long-term monitoring protocols, (3) integrated thermal–structural–hydraulic performance models, and (4) context-driven selection methodologies. By positioning this critical synthesis after the descriptive foundation, this review establishes a clear analytical framework that guides the subsequent discussion of biosorbent integration (Section 3) and phytoremediation synergies (Section 5). Following a systematic review of substrate components and performance metrics, Section 3 concludes with a critical synthesis of current research gaps to guide next-generation design.

## 4. Sorption Capacity and Substrate

As previously mentioned, the substrate acts as a synthetic soil on green roofs, and is therefore essential for the effective development of species and, consequently, for the success of the project. This parameter is of significant importance since it corresponds to the layer with the largest proportion of the green roof, and therefore several guidelines must be evaluated to choose an ideal substrate. In their totality, these parameters correspond to high stability, minimum apparent density dry and wet, the ability to firmly anchor plants, high water retention capacity, high efficiency in retaining nutrients, high porosity, air-packed, support for many different plant species, negligible organic components and high hydraulic conductivity. Due to the complexity inherent in each of these parameters, it is very difficult to build a green roof with all of these properties mentioned using a single component.

While guidelines from FLL and ASTM recommend a predominantly inorganic fraction (>80%) to minimize structural load, prevent weed invasion, and maintain high hydraulic conductivity, relying exclusively on inorganic aggregates creates significant functional limitations. Specifically, the absence of organic matter severely restricts nutrient availability, cation exchange capacity, and rhizospheric microbial activity, often stunting plant development and necessitating synthetic fertilizer applications [12,190]. These fertilizers, however, frequently leach into runoff, elevating concentrations of copper, phosphorus, and dissolved solids that degrade downstream water quality [191]. Furthermore, conventional inorganic materials exhibit inherently low sorption capacities for heavy metals and nutrients, limiting their ability to mitigate urban runoff contamination. To reconcile these structural requirements with ecological and water quality objectives, a strategic blend of inorganic and organic compounds is recommended. This hybrid approach leverages the lightweight, drainage-promoting properties of inorganic aggregates while utilizing organic fractions and biosorbents to enhance nutrient cycling, improve pollutant retention, and support robust vegetation growth without compromising hydraulic performance or substrate stability. As highlighted in the following sections, the integration of high-capacity biosorbents into this mixed matrix directly addresses the sorption limitations of traditional inorganic media while maintaining compliance with engineering weight and porosity thresholds.

As already highlighted, the parameters high air-filled porosity, water retention capacity, high hydraulic conductivity, and apparent density should present low limits, being favorable for most commercial green roof substrates. Based on the zero or minimum values of organic compounds, good stability, and plant support are not considered. In addition, the use of fertilizer is common when using commercial substrate, the main negative point is the worsening of the quality of the runoff. This corroborates a study that detected high levels of copper and phosphorus much higher after the addition of fertilizer [192]. One way to reduce undesirable contaminants is to add sorbents to the substrate fraction. It is worth noting that unfortunately inorganic materials generally have a lower sorption capacity for several chemical elements. Therefore, it would be necessary to add organic materials to increase sorption. For this purpose, the literature presents several organic materials with good adsorption capacities for several pollutants, including heavy metals [141,143,193]. In this same line of thought, researchers added coconut peat to the organic fraction on green roofs, where it was discovered (precipitation event with metal peaks of 70 mm) the retention of 66.6, 7.7, 15.1, 15, 68.1, 13, 8.8, and 16.4 mg for the metals aluminum, cadmium, chromium, copper, iron, nickel, lead, and zinc, respectively [60]. The main components of the inorganic fraction are vermiculite, crushed brick, slag, expanded clay, sand, heat-expanded slate, recycled glass, pumice stone, and recycled glass, as shown in Table 6. Unfortunately, these materials have low or no sorption capacity for several anions and cations. A study analyzed whether the inorganic compound slag had the potential to be used as an adsorbent in the adsorption of divalent metals present in the substrate, such as zinc, cadmium, lead, and copper [194]. Surprisingly, the slag removed all metals, however, with very low capacities, the maximum being 6.9 mg g^−1^ for lead and the minimum being 1.48 mg g^−1^ for zinc, while copper and cadmium presented values of 1.77 and 2.41 mg g^−1^, respectively. Pumice was also analyzed in another study where the removal of chromium was 1.6 mg g^−1^ and that of copper was 3.5 mg g^−1^ [195]. The best values were obtained in a study that analyzed perlite where the adsorption values for lead were 26.9 mg g^−1^; however, for cadmium, copper, and nickel, the values were low, being 2.81, 5.66, and 3.34 mg g^−1^, respectively [196].

Table 6 summarizes some relevant results on the removal of pollutants from the surface of inorganic materials. In general, the adsorption values are poor, and these materials, when compared with other adsorbents present in the literature for the same purpose, present no adsorptive competition, and are not recommended. To try to overcome this limitation, other organic compounds should be added due to their attractive functional group characteristics, and good textural properties will increase the sorption capacity of the substrate, improving the quality of the flow and reducing pollution. In addition, inorganic materials present chemical groups that also contribute to worsening the flow, an example is vermiculite, which presents oxides (SiO_2_, Fe_2_O_3_, and Al_2_O_3_) that are easily leached [197]. The inorganic substrates exhibited relatively low K_d_ values (0.02–35.29 L g^−1^), reflecting limited affinity for heavy metals under the tested conditions. Vermiculite showed the highest K_d_ for Pb(II) (35.29 L g^−1^), consistent with its elevated cation exchange capacity (41.3 meq 100 g^−1^). However, the leaching of structural oxides (e.g., SiO_2_, Fe_2_O_3_, Al_2_O_3_) from materials such as vermiculite may compromise effluent quality [197], highlighting a trade-off between sorption performance and chemical stability in inorganic amendments.

The improvement of plant anchorage, water retention, substrate stability, and nutrient potential for plants are high with the organic fraction. Table 7 shows this fraction and the types most used in green roofs. It can be noted that the most used include peat, coconut fiber, bark, vegetable composts, and mulch, which generally have a higher adsorption potential when compared to inorganic ones. A study analyzed mulch (pine bark nuggets, hardwood bark, and cypress bark) against the adsorption of zinc, copper, and lead [201,202]. The removal results were 12.2, 22.8, and 72.5 mg g^−1^ for the heavy metals zinc, copper, and lead, respectively. These results were the best and resulted from the use of residual wood bark present in the substrate. In another study, coconut pith was analyzed and the adsorption potential for the metals chromium, nickel, and cobalt was discovered, with capacities of 11.6, 16, and 12.8 mg g^−1^, respectively [203,204]. Organic amendments displayed moderate K_d_ values (0.17–19.17 L g^−1^), with hardwood mulch and green waste compost showing the highest affinities for Pb(II). Chemical modification (e.g., acrylic acid grafting on coir pith) enhanced both K_d_ and adsorption capacity, confirming that surface functionalization can improve metal binding [205]. Although organic substrates generally outperformed inorganic materials, their capacities remain insufficient for treating complex, multi-metal urban runoff without further optimization or combination with high-affinity biosorbents.

**Figure 6:**
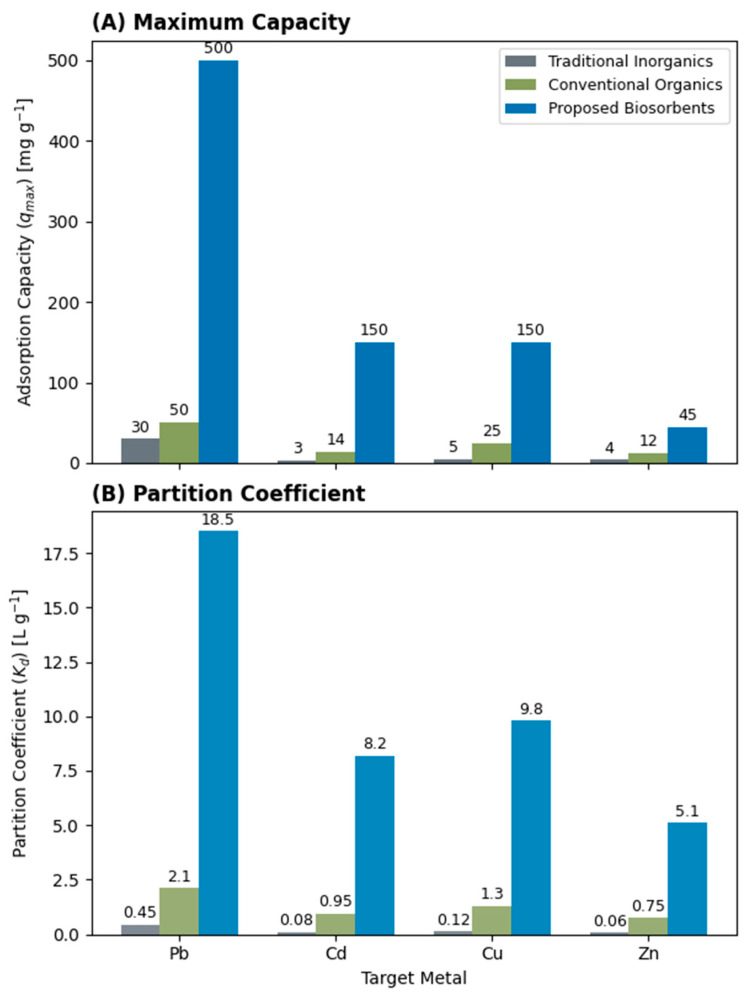
Comparative heavy metal retention performance across substrate types. (**A**) Maximum adsorption capacity and (**B**) partition coefficient for Pb, Cd, Cu, and Zn. Values are compiled from Table 6 and Table 7.

These capacities are not very high; however, they are average and much higher than those of inorganic compounds. Despite this, it is recommended to use an organic adsorbent with high adsorption capacities, which must be achieved without compromising other desirable properties. Figure 6 highlights the superior performance of the proposed biosorbents (seaweed, biochar, crab shell) relative to conventional inorganic and organic substrates. The marked contrast in both adsorption capacity and K_d_ underscores the value of waste-derived, high-affinity materials for multi-pollutant retention. Integrating these biosorbents into substrate formulations enables a complementary retention strategy, combining ion exchange, complexation, and redox mechanisms, that aligns with circular economy principles while addressing the sorption limitations of standard green roof media. While conventional inorganic aggregates and organic amendments exhibit limited adsorption capacities, often insufficient to address the complex pollutant load of urban runoff, the proposed biosorbents demonstrate a marked superiority. This quantitative contrast underscores the necessity of shifting from traditional substrate formulations toward high-performance, waste-derived additives. Integrating these biosorbents not only addresses the sorption limitations of standard materials but also aligns with circular economy principles by valorizing low-cost biomasses.

### 4.1. Biosorbents

Before detailing the mechanistic foundations of biosorption, it is essential to contextualize the practical efficacy of the three strategic biosorbents evaluated in this review. Synthesizing data from Table 6, Table 7 and Table 8 and the source literature, the following removal efficiency ranges are reported under conditions relevant to green roof runoff. Biochar, when integrated into green roof substrates at 5–10% (*v*/*v*), biochar has demonstrated 47–86% removal of total phosphorus and nitrate, up to 86% reduction in total suspended solids, and 17–75% removal of heavy metals (Zn, Cd, Ni, Cr, Pb, Cu) depending on pyrolysis temperature, feedstock, and initial pollutant concentration [209,210]. Higher efficiencies (>70%) are typically achieved for cationic metals (Pb^2+^, Cu^2+^) at pH 5–6, where surface complexation and electrostatic attraction dominate. Seaweed biomass (brown, green, red algae), batch and column studies report 66–88% retention of cationic metals (Al, Cd, Cr, Cu, Fe, Ni, Pb, Zn) under simulated rainfall events with metal peaks of ~70 mm [211,212]. In packed-column configurations mimicking green roof drainage, brown algae (e.g., *Sargassum* spp.) maintained >80% copper removal over 21–41 days of continuous operation [213,214]. Efficiency is pH-dependent, with optimal performance at pH 4–6 for cationic species; anion removal (e.g., phosphate) reaches ~60 mg g^−1^ capacity but is more sensitive to competing ions in complex matrices [215]. Raw crab shells exhibit exceptional affinity for lead and phosphate via CaCO_3_-driven microprecipitation, with reported removal efficiencies >90% for Pb^2+^ and ~85% for PO_4_^3−^ in batch tests at pH 4–6 [216,217]. For other metals (Cd, Cu, Co), efficiencies range from 70 to 95% depending on initial concentration and contact time [218,219]. In column studies, crab shell beds maintained >80% nickel removal over multiple cycles, though regeneration requires acidic eluents that may not be feasible for passive green roof systems [220].

It is critical to note that these efficiencies are context-dependent: batch studies with synthetic single-metal solutions typically report higher values than column or real-wastewater experiments due to the absence of competing ions, variable pH, and dynamic flow conditions. For green roof applications, characterized by intermittent rainfall, multi-pollutant loads, and fluctuating pH, the lower end of these ranges should be used for conservative design. The following subsections detail the mechanisms underpinning these performances and strategies to optimize efficacy under realistic conditions.

Before detailing the specific adsorptive functions of individual materials, it is essential to clarify the typicality and strategic rationale behind selecting biochar, seaweed biomass, and chitin-rich crab shell waste as the focal biosorbents in this review. These three materials were intentionally chosen because they represent distinct, complementary classes of waste-derived biomaterials that collectively encompass the primary mechanisms relevant to green roof runoff remediation: (i) biochar typifies carbonaceous, high-surface-area amendments that dominate adsorption through surface complexation, electrostatic attraction, and ion exchange; (ii) seaweed biomass represents polysaccharide-rich marine macroalgae that leverage alginic acid, fucoidan, and sulfate/carboxyl groups for efficient cation binding and exceptional water retention; and (iii) crab shell waste exemplifies chitin- and calcium carbonate-rich crustacean residues that facilitate metal and phosphate removal via microprecipitation, pH buffering, and chelation. Beyond their mechanistic diversity, these biosorbents are globally abundant, low-cost, and align directly with circular economy objectives by valorizing agricultural, marine, and seafood processing wastes. Critically, their physicochemical properties are highly compatible with green roof substrate engineering, as they can be integrated at controlled dosages to enhance pollutant retention, nutrient cycling, and drought resilience without compromising the lightweight, high-conductivity requirements mandated by established guidelines (FLL, ASTM). By focusing on these three representative materials, this review provides a comprehensive and transferable framework for evaluating biosorption performance that can be extrapolated to other waste-derived amendments in urban stormwater management systems [221,222,223]. The functional groups and their charges and the textural properties (such as surface area and porosity) influence the values of adsorption capacity. In addition, there are also experimental conditions, since adsorption is also strongly influenced by the pH value and temperature [224,225,226]. Among the interaction mechanisms, the literature highlights microprecipitation, ion exchange, chelation, electrostatic interaction, coordination, complexation, dipole–dipole, hydrophobic interactions, and hydrogen bonds [227,228]. Most interactions are physical since they have lower specificity and require less energy. The group of biosorbent materials described in the literature is very extensive; these materials generally originate from agricultural waste, algae, industrial waste, bacteria, and fungi. Since the focus of this review is on materials with the potential to be used as green roof substrates, they must provide support for plant development. Therefore, the basic properties present in the organic fraction must be preserved. The recommendation corresponds to organic materials composed of seaweed, biochar, and seafood waste, in the specific case of crab shell residue. Working with materials that are abundant in nature and low cost, such as waste, is important because it corroborates the problem of pollution, brings cost–benefit, and increases the sustainability of the process [229,230,231]. Detailed analyses of the adsorptive performance of these materials are provided below.

#### 4.1.1. Seaweed

Seaweeds are renewable biomasses found naturally in the environment. They can multiply rapidly and are found in large volumes in oceanic aquatic environments. The use of these organisms is very variable, including livestock nutrition [232], human consumption [233], and fertilizer [234]. These aquatic organisms also release important phycocolloids such as carrageenan, alginic acid, and agar [235]. Regarding the adsorption potential, seaweeds have good remediation capacity, where the main group of adsorbates present in the literature are heavy metals [236,237]. The classification of the various groups of algae can occur by the type of flagellation, cell wall chemistry, and chlorophyll nature. Due to these characteristics, they are divided into eight groups, namely *Charophyta*, *Phaeophyta*, *Cyanophyta*, *Chlorophyta*, *Euglenophyta*, *Rhodophyta*, *Cryptophyta*, and *Chrysophyta* [236]. The most commercially important and most common divisions are brown algae (*Phaeophyta*), red algae (*Rhodophyta*), and green algae (*Chlorophyta*). Both have as typical cell walls a fibrillar skeleton that contains mostly cellulose; however, to a lesser extent, it may present only xylan in the case of red algae, or only mannan or xylan in the case of green algae. These algae also have a matrix with regular or uniform incorporation that contains mostly alginic acid or alginate in addition to smaller amounts of fucoidan (sulfated polysaccharide) present in brown algae. Sulfated galactans occur in large quantities in red algae [211].

Regarding the adsorptive performance, brown algae showed better potential, which may be related to their specific chemical composition [236,237]. In this sense, three species of brown algae (*Sargassum fluitans*, *Ascophyllum* *nodosum*, and *Fucus vesiculosus*) were used to remove lead [211]. The values of adsorption capacities were excellent, being 330, 478, and 600 mg g^−1^ for the species *Sargassum fluitans*, *Ascophyllum nodosum*, and *Fucus vesiculosus*, respectively. Table 8 describes the performance of the group of brown algae and their favorable interaction with various types of chemical pollutants. Alginic acid, also known as a carboxylated copolymer, presents a linear arrangement with variable amounts of β-d-mannuronic acid, which is linked to α-l-guluronic acid (G block) and 1,4 (M block). In brown algae, this copolymer corresponds to approximately 10 to 40% of its dry weight [236]. When analyzing the sequence of the G and M blocks, very different structures are observed. In the case of the proportion contained in these blocks of alginic acid, it interferes with the physical properties and reactivity of the polysaccharide [237]. Carboxyl groups are abundant in alginate, in addition to fucoidan sulfonate groups and hydroxyls. Both three have critical functions in adsorption; the level of importance and adsorption will depend on the pH value, the type of ion, the environmental conditions, and the charge of the ion. When pH values are low, it favors the protonation of chemical elements with positive hydrogens or with other groups of light metal ions, which also corroborates the occupation of functional groups [228,236]. Raising pH values to a more alkaline medium modifies the surface charges present on the surface of the algae, increasing the negativity. This is due to the reduction in the concentration of positive hydrogens, corroborated by a greater interaction with cationic adsorbates. In general, adsorption was favorable in several studies for a pH range between 4 and 6, which is a slightly acidic to neutral condition, when it comes to the remediation of cationic species. Since precipitation water is usually between these values, it is assumed that the biosorbent has good remediation potential.

For the desorption step, when the adsorbate is removed from the surface of the adsorbent, acidic chemical reagents are used. These desorbents can also be called eluents; in this sense, a study used various eluents in order to recover cobalt from the surface of the species *Ascophyllum nodosum* [238]. The eluents analyzed were NH_4_OH, CaCl_2_, KSCN, ethylenediaminetetracetic acid, KHCO_3_, HCl, KCl, and H_2_SO_4_. The efficient desorption of 96% was only possible using a solution of 0.05 M CaCl_2_ in hydrochloric acid with an acidic pH of 3 as eluent. This confirms that the surface of the algal biomass is strongly interacting with the metal ions; therefore, it is assumed that the ions will be difficult to leach via flow. The plant’s phytoremediation potential also allows for the removal of pollutants present in the substrate from the system (a topic discussed in the following sections). Contaminated solutions can also be efficiently purified by brown algae [213,236]. The potential of brown algae to continuously adsorb pollutants was analyzed using packed column arrangements [239]. The arrangement of a vegetative roof is similar to a packed column, so the results obtained may be useful for this investigative review. A packed bed column with *Sargassum filipendula* biomass showed that copper adsorption occurs over a period of 41 days [213]. In another study, a packed column with *Turbinaria ornata* operated for 21 days with continuous copper adsorption [214]. In situations of real water contamination, it is possible to observe the presence of several metal ions, which increases the chances of competitive adsorption, causing competition to occur to reach the adsorptive sites present on the surface of the biosorbent [240,241,242,243].

In order to understand how this competition occurs, it is vital to conduct studies with more complex solutions and not only with unitary or binary solutions. Statistical physical modeling and density functional theory studies also help in understanding these interactions and the energies that accompany them [244,245,246]. Real wastewater was treated using a hybrid packed column containing *Sargassum* species and sand [247]. The experimental conditions were as follows: pH value of 1.1, conductivity value of 6.98 mS cm^−1^, salinity of 3.77 and total dissolved solids of 4.46 g L^−1^. The wastewater contained about 14 metals (Zn, Al, Pb, Ca, Ni, Cd, Na, Co, Mn, Cr, Mg, Cu, K, and Fe), and it was observed that part of these metal ions were removed efficiently; however, under extreme conditions of pH values, the removal of other metal ions was affected. Seaweeds had their adsorptive performance severely affected under conditions of low pH, high salinity, high conductivity, and total dissolved solids [34]. A packed column with Sargassum was used to treat complex electroplating wastewater, where the nickel content was 109 mg L^−1^ with a total hardness of 580 mg L^−1^ and a total dissolved solid of 1489 mg L^−1^ [212]. The adsorption of nickel ions was good at 21.7 mg g^−1^, with the potential for efficient regeneration for up to five desorption cycles.

**Table 8:** Organic sorbents with potential for use as green roof substrate and their respective adsorptive capacities.

Anion/Cation	Adsorbent	Desorption Capacity (mg g^−1^)	Experimental Conditions	Reference
PO_4_	*Kappaphycus alvarezii*	60	Batch; Synthetic single anion; pH 5–6; C_0_ = 50 mg L^−1^	[215]
PO_4_	Biochar (*Thalia dealbata* at 700 °C)	5	Batch; Synthetic single anion; pH 7; C_0_ = 40 mg L^−1^	[248]
Cr (III)	*Chinonecetes opilio*	55.1	Batch; Synthetic single metal; pH 4–5; C_0_ = 60 mg L^−1^	[249]
Pb	*Portunus trituberculatus*	870	Batch; Synthetic single metal; pH 5; C_0_ = 100 mg L^−1^	[216]
Ni	*Kappaphycus alvarezii*	22.3	Batch; Synthetic single metal; pH 5–6; C_0_ = 50–70 mg L^−1^	[250]
F	Biochar (spent mushroom compost coated with Al(OH)_3_)	36.5	Batch; Synthetic single anion; pH 6–7; C_0_ = 100–120 mg L^−1^	[251]
Cr (III)	*Ulva Lactuca*	150	Batch; Synthetic single metal; pH 4–5; C_0_ = 10–50 mg L^−1^	[196]
PO_4_	Crab shell	109	Batch; Synthetic single anion; pH 6; C_0_ = 100–200 mg L^−1^	[217]
Pb	*Gracilaria corticate*	50	Batch; Synthetic single metal; pH 5; C_0_ = 60 mg L^−1^	[252]
Pb	*Kappaphycus alvarezii*	105.7	Batch; Synthetic single metal; pH 5–6; C_0_ = 30–80 mg L^−1^	[250]
Cd	*Ulva Lactuca*	43	Batch; Synthetic single metal; pH 5; C_0_ = 120–200 mg L^−1^	[253]
Pb	Biochar	29	Batch; Synthetic single metal; pH 5–6; C_0_ = 55–120 mg L^−1^	[254]
Zn	*Ascophyllum nodosum*	42	Batch; Synthetic single metal; pH 5; C_0_ = 10–160 mg L^−1^	[255]
Cd	Biochar (*Canna indica* at 500 °C)	188.8	Batch; Synthetic single metal; pH 6; C_0_ = 150 mg L^−1^	[256]
Cr (VI)	*Ucides cordatus*	28.1	Batch; Synthetic single metal; pH 2–3; C_0_ = 50–260 mg L^−1^	[218]
Cd	*Crab shell (Chinonecetes opilio)*	199	Batch; Synthetic single metal; pH 5; C_0_ = 60–160 mg L^−1^	[257]
Pb	*Sargassum fluitans*	330	Batch; Synthetic single metal; pH 4–5; C_0_ = 130 mg L^−1^	[211]
Pb	*Ulva Lactuca*	125	Batch; Synthetic single metal; pH 5; C_0_ = 20–120 mg L^−1^	[252]
Cd	*Kappaphycus alvarezii*	54	Batch; Synthetic single metal; pH 5–6; C_0_ = 30–180 mg L^−1^	[250]
Cu	*Ascophyllum nodosum*	59	Batch; Synthetic single metal; pH 5; C_0_ = 90 mg L^−1^	[255]
NH_4_	Biochar (*Thalia dealbata* at 700 °C)	17.6	Batch; Synthetic single cation; pH 7; C_0_ = 60 mg L^−1^	[248]
Cd	*Ascophyllum nodosum*	215	Batch; Synthetic single metal; pH 5; C_0_ = 50–150 mg L^−1^	[258]
Pb	*Sargassum natans*	224	Batch; Synthetic single metal; pH 4–5; C_0_ = 120 mg L^−1^	[252]
Pb	*Ascophyllum nodosum*	360	Batch; Synthetic single metal; pH 4–5; C_0_ = 50–150 mg L^−1^	[211]
Cu	*Portunus sanguinolentus*	244	Batch; Synthetic single metal; pH 5; C_0_ = 60 mg L^−1^	[219]
Mn	*Ulva Lactuca*	58.8	Batch; Synthetic single metal; pH 5; C_0_ = 300 mg L^−1^	[196]
Ni	*Ascophyllum nodosum*	43.3	Batch; Synthetic single metal; pH 5; C_0_ = 60 mg L^−1^	[255]
Co	*Portunus trituberculatus*	322.6	Batch; Synthetic single metal; pH 5; C_0_ = 100 mg L^−1^	[219]
Cu(II), Ni(II), Zn(II)	*Sargassum* sp. + sand (hybrid column)	21.7 (Ni)	Column; Real electroplating wastewater; pH 1.1; 5-cycle regeneration	[212]
Pb(II), Cd(II), Cu(II)	*Sargassum filipendula* (packed column)	41-day breakthrough	Column; Synthetic multi-metal; pH 4; continuous flow	[213]
Cu(II), Zn(II)	*Turbinaria ornata* (packed column)	21-day operation	Column; Synthetic binary; pH 4.5; continuous flow	[214]
Pb(II), Cd(II), Cu(II), Zn(II)	Crab shell (raw)	1.5–6.9	Batch; Synthetic quaternary mixture; pH 4–5; competitive conditions	[219]

In addition to brown algae, green algae also have good potential for removing anions and cations [259,260]. The metals Cr(III) and Mn(II) showed capacities according to the Langmuir monolayer model [261] of 150 and 58 mg g^−1^, respectively, with the use of the biomass of the algae *Ulva* sp. [196]. The cell wall of the algae contains approximately 38 to 54% polysaccharides on a dry-weight basis. In addition, the wall is composed of ulvan, an important polyelectrolyte with a high affinity for metal cations, precisely because it contains sulfate and carboxylic groups in its structure. Table 7 also presents other adsorption values achieved with green algae, where it is possible to observe good efficiency in real wastewater [228], in multicomponent solutions [262,263], in continuous experiments [264] and in multicomponent solutions [262,263]. In the same line of research, red algae also showed good performance in experiments with wastewater treatment modules [265], in column operation modes [266], and in batch operation modes [215] for metal remediation. Due to the extensive research analysis to resolve the biosorption potential of seaweeds, especially in the last 20 years, it is very difficult and complex to indicate seaweeds of practical use and with high efficiency in the application as biosorbent additives in green roofs.

Increased water retention via swelling and seaweed absorption provides benefits from adding seaweed biomass to green roof substrates. One study found that the species *Sargassum* sp. presented a 260% water absorption capacity, while 170% was the value obtained by the species *Turbinaria conoides* [267]. The species *Sargassum wightii* also obtained a high swelling capacity with 10 mL g^−1^ of dry weight [268]. In biosorption techniques, this property can be a negative point, since swelling ends up hindering the flow of water, increasing the pressure in the system, and possibly causing the interruption of the columns used in wastewater remediation. The presence of large volumes of inorganic materials in green roofs is also responsible for the increase in hydraulic conductivity. For example, crushed bricks that are widely used in green roof substrates present 14,200 mm/h of hydraulic conductivity [269]. This has adverse effects on the potential for reducing peak rainwater flow, for these situations’ algae with swelling potential and retention, can corroborate by adjusting the hydraulic conductivity present excessively. Algae are also used to accelerate plant growth, presenting properties of nutritional additives, biostimulants, and biofertilizers for the soil. In usual studies, green roofs present the need for the addition of chemical fertilizers, increasing contamination due to the reduction in the surface layer of the soil and other toxic elements that are released in the runoff [32,270].

Due to the increase in runoff water contamination, mainly due to fertilizers, the application of natural organic additives such as algae is recommended and highly viable, as they will contribute to soil structure and plant growth. These aquatic organisms contain amino acids and provide micro and macronutrients and metabolic improvements [271]. The alginates, auxins, and cytokines present in algae are plant growth activators and sources of potassium [271]. Algae species used to correct soil nutrition include: *Ascophyllum nodosum*, *Durvillaea* sp., *Laminaria* sp. *Sargassum* sp., and *Ecklonia maxima*. Productivity and enhanced growth were observed by several researchers who used seaweed extracts as a compound [272]. There are also reports of improvements in the control of nematodes present in the soil and in combating pests such as insects [234]. Algae that act as biostimulants increase the tolerance of plants to biotic and abiotic stresses [273], raise the amount of chlorophyll in the leaves [274], improve the germination rate of seeds with delayed senescence, and finally, increased the useful life of the plant [275].

#### 4.1.2. Biochar

Biochar is a dark-colored solid compound rich in carbon, which is produced by pyrolysis in a limited oxygen atmosphere [229,246,276,277]. This product has been used to solve different environmental problems such as carbon mitigation (sequestration) [278], soil and water remediation by acting as an adsorbent [279] and improving soil fertility [47]. Due to these characteristics, its use in green roofs can improve the properties of the substrate, such as hydraulic conductivity [280], pH value [281], aeration [282], nutrient retention [283] and water retention capacity [284]. In addition to these advantages, it has economic benefits since it can be produced from materials present in very low or zero-cost stocks. Other scientists also recommend biochar in green roofs, claiming that it improves runoff quality [47,210] and reduces substrate weight [285]. As a substrate additive, it has a water retention potential ranging from 77 to 79% [47,286]. By adding approximately 7% biochar to a green roof substrate, the authors highlighted an increase in the quality of runoff water with a significant reduction in TOC, total nitrogen, turbidity, total phosphorus (TP), nitrate, and phosphorus [210]. Acting as a fertilizer, biochar acts as a slow-release carrier [287]. In this sense, it increases soil microbial activity and organic matter content [288,289]. This corroborates the results obtained in another study where biochar increased soil porosity, reduced apparent density, increased aeration, as well as volumetric water content and its retention [290].

With these results described above, the root system of plants may have a greater potential to fix oxygen due to the moisture made available, intensifying the growth of the individual. Regarding water remediation, it has the potential to remove several organic pollutants [243,291,292] and mainly metal ions [293,294], and other anions [295]. Weed residues were used to obtain biochars with different textural properties through pyrolysis at temperatures of 300, 500, and 700 °C [294]. The adsorbent carbonized at the highest temperature presented the best adsorption capacity, being 11.6 mg g^−1^ and 333.3 mg g^−1^ for the metal’s cadmium and lead, respectively. This may be related to the fact that the higher temperature provided a larger surface area and porosity with pores of strategic diameters for the adsorption of metal ions. In addition, unlike other organic adsorbates, heavy metals present a strong dependence on surface charges; therefore, the groups belonging to the carbonaceous material present opposite charges to the metals, corroborating the interaction and electrostatic attraction. The main mechanisms involved in the adsorption of anions and cations on the surface of biochar are precipitation, ion exchange, electrostatic interactions, and surface complexation [296]. The methodology for obtaining biochar is very variable since there are hundreds of processes in the literature that are diverse among themselves. This is because the aim is to obtain a final product with a good (high) surface area and strategic porosity with the adsorbate molecule to be removed (micro, meso, and macropores) [226,291]. A study that used biochar with favorable properties managed to reduce the TSS present in the runoff by up to 86%, while the concentration of nitrate and phosphorus was reduced by 86 and 47%, respectively [209]. In the same study, the authors reported the reduction in other metal ions ranging from 17 to 75% for the metals zinc, cadmium, nickel, chromium, lead, and copper, with the highest removal for copper and the lowest for nickel. Therefore, focusing on the development of a substrate designed to improve runoff water quality, mainly focusing on metals and nutrients, is highly viable for green roof engineering. Studies should not be focused solely on a pilot scale, and in these cases, the focus should be on analyzing the potential of these additives in real scenarios and over extended periods, aiming to analyze the interference of the seasons (dry and rainy; hot and cold).

#### 4.1.3. Crab Shell

Another group also used as a biosorbent and with a high potential to remove pollutants and heavy metals are the residual crab shells. The adsorptive potential is related to the main presence of chitin in addition to other elements such as magnesium, calcium (carbonates), and proteins [217,249,297]. Another issue is that seafood cooking results in a large volume of waste such as crab shells. The management of this waste as well as its disposal is a serious environmental problem. Therefore, recycling would be a highly sustainable means in addition to generating profits for the seafood market. This is a serious problem since current reports estimate that crab shells generate millions of tons of waste annually worldwide. In terms of numbers, it is estimated that between 5000 and 8000 tons are discarded by the seafood industry alone, not taking into account the individual activities of small traders, such as the riverside population [218]. Due to this problem, several researchers have also begun to analyze its use as a source of chitin and adsorbent [297,298], as a fertilizer for crops [299] and as a soil conditioner [300].

The adsorption properties are influenced by the presence of calcium carbonate; in the case of metals, these are microprecipitates [297]. Calcium carbonate dissociates with metal ions; this process is influenced by pH. Individually, the carbonate and calcium ions come into contact with the metal where precipitates of metallic carbonate are generated and absorbed by the chitin that makes up the crab shell. In one study, raw crab shell particles were responsible for removing lead from water [216]. The authors state that the mechanism occurred because the metal bound to CO_3_^2−^ and –NHCOCH_3_ through the dissolution of calcium carbonate leading to the precipitation of PbCO_3_ and Pb_3_(CO_3_)_2_(OH)_2_. Other studies have shown the potential of this biomass in the removal of other metals, such as arsenic [301], lead [297], cobalt [219], chromium [302] and copper [219]. In addition to batch experiments, it is also possible to observe studies that analyzed the efficiency of remediating metals in a packed column system, where the column filled with crab shells efficiently removed nickel with good desorption potential [220]. It is important to emphasize that the use of raw shells does not make the desorption or regeneration process a viable alternative, since removing the adsorbate from the surface of the solid requires the use of eluents and other energy consumption costs. Therefore, these studies should only be carried out in cases where complex and expensive adsorbents are used, since desorption also generates the production of a secondary source of effluent, which cannot be discarded without prior treatment back into the water compartments [229,244]. Solutions containing another pollutant were also effectively remediated by crab shells [302], in addition to real wastewater [214,303].

When used as a soil additive, the shell has great potential for organic fertilization, supporting growth by releasing nutrients such as phosphorus and nitrogen, increasing the organic and physical properties of the soil [300]. This limits the need for expensive inorganic fertilizers in green roofs. Chitin can control pathogens present in the root system, supporting the reproduction of rhizobia, and increasing nitrogen fixation (symbiosis). In a study, it was observed that the total available phosphorus, the exchangeable magnesium and calcium content, and the pH increased in acidic soils after the addition of crab shell powder [224,299]. Growth at maturity and soybean seed productivity were increased in the area of soil modified with crab shells [300]. Due to these properties, it is indicated that modifying soils with this residue can bring significant results in soybean productivity and can be competitive with NPK fertilizers, which are frequently used. This would also be in line with possible applications in green roofs; however, pilot tests should be conducted analyzing various plant species and under varying precipitation and temperature conditions.

### 4.2. Critical Engineering Trade-Offs and Practical Implementation Constraints

While the adsorption capacities of biosorbents significantly outperform conventional substrate components, their integration into green roof systems introduces critical engineering trade-offs that must be carefully balanced to maintain system functionality. The high swelling capacity of seaweed biomass, for instance, enhances water retention but concurrently reduces saturated hydraulic conductivity. Under intense or prolonged rainfall events, excessive swelling can lead to pore clogging, surface ponding, and the development of localized anaerobic conditions. These conditions may compromise root respiration, promote denitrification (potentially increasing N_2_O emissions), and accelerate the mineralization of organic fractions, ultimately reducing long-term substrate stability. To mitigate hydraulic impairment, seaweed amendments should be limited to 5–10% by volume, pre-processed to control particle size distribution, and homogeneously blended with high-conductivity inorganic aggregates (e.g., expanded shale, pumice, or crushed brick) to maintain hydraulic conductivity above the FLL-recommended threshold of 3600 mm/h. Biochar, despite its exceptional porosity and surface area, presents initial hydrophobicity that can temporarily delay water infiltration until surface wetting occurs. Furthermore, highly alkaline biochars may elevate substrate pH beyond optimal ranges for many green roof species, while low-temperature pyrolyzed biochars risk leaching soluble organic carbon or loosely bound nutrients during early rainfall events. Pre-wetting, thermal aging, or co-composting prior to substrate incorporation can effectively neutralize these effects and stabilize surface chemistry [126].

Crab shell waste relies on CaCO_3_ dissolution for microprecipitation and pH buffering, but this process gradually degrades the structural matrix of the amendment. Long-term dissolution may reduce substrate load-bearing capacity, increase calcium and alkalinity leaching, and potentially saturate adsorption sites, necessitating periodic replacement or regeneration in intensive systems. Particle size optimization (typically 1–4 mm) and blending with stable mineral matrices are essential to preserve structural integrity over multi-year dry–wet cycles. Collectively, these trade-offs underscore that biosorbent efficacy cannot be evaluated solely by maximum adsorption capacity. Successful implementation requires a systems-level approach that accounts for intermittent hydrological loading, pH dynamics, and long-term material degradation. Future pilot-scale studies must prioritize coupled hydraulic–chemical monitoring to establish optimal amendment ratios, identify critical thresholds for hydraulic conductivity loss, and develop maintenance protocols that sustain both pollutant retention and plant viability over the operational lifespan of green roof systems [304].

## 5. Phytoremediation and Plant Sorption Potential

The quality of water runoff in green roofs can also be improved by using plants that have phytoremediation potential. This would reduce the concentration of metals, increasing the quality of the runoff, since lower concentrations would leach into this effluent [305]. This process consists of the direct use of microorganisms and plants that are related and present in a given soil to reduce the contamination present in these areas, which is caused by contaminated water (via human activity) [306,307]. This process occurs via four independent steps, namely: phytoextraction, rhizofiltration, phytovolatilization, and phytostabilization. Rhizofiltration remediates water through roots that are kept alive hydroponically, which can precipitate, adsorb, and concentrate contaminants [308]. In the case of phytostabilization, the plant is responsible for stabilizing the contaminated soil, not causing direct removal or cleaning. The consumption of contaminants via the plant’s metabolic system occurs in phytovolatilization, and finally, in phytoextraction, the pollutants are consumed via the root system, moving them to the aerial fractions via the xylem. The species most recommended for phytoremediation are those that have a high phytoextraction potential and are therefore recommended for application on green roofs, increasing runoff efficiency [309]. It is worth noting that no studies have currently been found that analyzed this property when selecting plant species for green roofs. Table 8 shows some plant species with phytoextraction potential that can be used in future studies on green roofs. To facilitate the screening of species for phytoremediation applications, Table 9 includes the bioconcentration factor (BCF = C_root_/C_substrate_) and translocation factor (TF = C_shoot_/C_root_) where reported in the source literature. Species exhibiting BCF > 1 and TF > 1 are considered strong candidates for phytoextraction, as they efficiently accumulate metals in roots and translocate them to harvestable aerial biomass. For entries marked “–”, substrate concentrations were not reported in the original study, preventing BCF calculation; however, readers may estimate BCF if substrate data become available. TF values were calculated from reported tissue concentrations when shoot and root data were provided.

**Figure 7:**
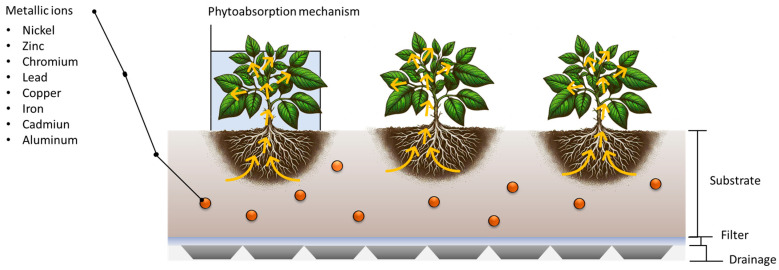
Phytoextraction process of metals present in the substrate of vegetal roofs.

The crucial point of the phytoextraction process is the species’ ability to accumulate metals in its cells present in the root system. However, this process is only efficient if the adsorbed metal can be transported to the aerial parts so that it can be removed from the system and does not generate toxicity to the plant, which can lead to its death [318]. Only with this process is it possible to obtain long-term and favorable benefits in green roofs, since there is a need to remove these ions from the atmosphere. Figure 7 illustrates possible mechanisms that are most common for the phytoextraction of metals in the substrate in green roofs. Translocation controls the movement of metals that enter via the root under pressure and go to the stem so that transpiration occurs through the stomata [319,320]. Physiological barriers can occur in the roots, corroborating the accumulation in the root system since translocation is prevented, while other metals can be easily translocated.

Plants, like humans, need some metals to survive. These are divided into micro and macro depending on their level of importance. Among them, calcium, zinc, copper, nickel, iron, and potassium stand out. However, other metals such as cadmium, aluminum, lead, and chromium do not act in any metabolic pathway. Despite this, it has been shown that some species accumulate these metals or vital metals through the phytoextraction mechanism. These individuals are also called hyperaccumulators [321]. These individuals encompass a special category that comprises approximately 45 families and 400 species [322]. This group may be a viable alternative for remediating soils present in green roof substrates through mechanisms such as mineralization, adsorption, transformation or hyperaccumulation, translocation, and transport of metals [323]. Unlike other species, these pollutants would not cause cell damage or possible adverse effects of toxicity. The capacity to accumulate metals is about a hundred times greater than that of average plants. The species *Arabis paniculata* was analyzed for its hyperaccumulation potential against zinc, cadmium, and lead [324]. The authors confirmed that the plant was hyper-tolerant, with higher accumulation in the shoots with values of 434, 2300, and 20.800 mg/kg for the metal’s cadmium, lead, and zinc, respectively. It was also observed that these values were only possible because the translocation factors (TF) were above one. The *Sedum* species (popularly used in green roofs) also showed potential to hyperaccumulate metals in a study. This study found that *Sedum alfredii*, present in mining regions of China, has the potential to hyperaccumulate zinc [325,326] and cadmium [327]. Therefore, it is highly recommended that the selection of the species used in a green roof have either hyperaccumulation or phytoextraction potential, which would corroborate a potential improvement in the quality of the runoff. Finally, it is worth noting that hyperaccumulators should preferably present a bioconcentration and TF greater than one [328].

The bioconcentration factor (BCF) determines the plant’s potential to accumulate metal in the roots from the substrate. It can be calculated by the ratio between C_root_ (concentration of the metal present in the roots when harvested, mg kg^−1^) and C_substrate_ (representing the concentration of the metal initially measured in the substrate, mg kg^−1^) [328]. The TF indicates the plant’s potential to translocate the metal from the root region to the shoots. It can be calculated by the ratio between C_shoot_ and C_root._ The nature of the metal will interfere with the phytoextraction property of the plants [307]. The presence of metals in the substrate occurs in the form of a fluctuating equilibrium, which is generally controlled by the substrate. Of all the metals present in the soil, only a fraction is available for plants to use, this is because most are insoluble and cannot be used by plants [329]. The potential of the plant in terms of phytoextraction can be affected by the high permanent concentration of metals; with this, the bioavailability of the ions becomes a critical parameter for the subsequent accumulation in the plant tissues. Metalloids and heavy metals are divided into three groups; these groups refer to the bioavailability of the ions in the soil [330]. Arsenic, cadmium, copper, selenium, zinc, and nickel comprise the group of widely available metals. The group of slightly available metals comprises cobalt, iron, and manganese, and the slightly available metals include chromium, lead, and uranium. Phytosiderophore material is produced in the roots, which increases the efficiency of the metal mobilization stage and subsequent mobilization by the rhizosphere system [331]. In the case of grasses, avionic and mugineic acids, also called siderophores, are released. These can increase the availability of iron for subsequent absorption by the plant [332]. The presence of H^+^ ions in the roots causes the soil pH to acidify in the root region, which releases the metal cations present in the soil, corroborating the dissolution of the metals [333]. This process increases the concentration of metal ions in the solution [334]. Considering that the substrates of green roofs contain metals and inorganic and organic compounds, it is highly complex to determine which vegetation is efficient in remediating a mixture of metallic species.

The addition of chemical additives (ethylenediaminetetraacetic acid and limestone) has been used and has shown good results [307,335]. Despite this, their use is highly phytotoxic and can impoverish the soil precisely because it negatively affects beneficial microorganisms in the soil. These fertilizers can also easily leach into the final runoff. In this sense, this study sought to analyze methodologies that can be used and that present greater sustainability to the environment, which led to advanced processes mediated by microbes in which microbial processes and metabolites, both present in the root region, modify the mobility and bioavailability of metals. These mechanisms alter the potential for metal absorption by plant species [313,336]. Root growth is also stimulated by bacteria, which increases the individual’s resistance and development, making it more tolerant to environmental stresses such as lack of water. These microorganisms can support the phytoremediation of metals. Given the growing demand for remediation of contaminated environments, this technique has been the subject of study over the last ten years and has proven successful in several investigations [336]. Table 10 lists the species that have been used with various bacteria in phytoremediation techniques for areas contaminated by metals.

One study used the species *Pteris vittata* in conjunction with 5 types of arsenate-reducing bacteria (via manual supplementation) to increase metal absorption [337]. The microorganisms acted as a growth stimulant for the plant species; thus, the metal accumulated in the plant tissues and the insoluble arsenic present in the soil was activated. This process caused the leached arsenic to be significantly lower than that found in the untreated control test. The absorption of the metal by the plant was 44%, an important factor influenced by the increase in biomass by 53%. The presence of the reducers reduced leaching to 71% of 100%. In another study, the accumulation of the metal in the species *Sedum alfredii* was analyzed; this study used the bacterium *Burkholderia cepacia* [338]. Similar to the other study, the presence of the microorganism accelerated plant biomass, being 110% in the experiments with zinc, and the absorption in the shoots for the treatments with the metals zinc and cadmium increased by 96 and 243%, respectively. The microorganism also corroborated an increase in the translocation of metal in the root system to the shoots; this increase was 135 and 296% higher in the treatments with zinc and cadmium, respectively. The levels of tolerance of the plant to possible environmental stresses were increased by up to 134% in the experiment that had zinc added.

**Table 10:** Combinations of bacteria and plants with the potential for phytoremediation of metals.

Associated Bacteria/ Plant Species	Metals	Effect of Bacteria	Reference
*Variovorax* sp. SaNR1, *Burkholderia* sp. SaZR4, *Sphingomonas* sp. SaMR12 and *Burkholderia* sp. SaMR10/*Sedum alfredii*	Zn and Cd	*Burkholderia* sp. promoted Zn extraction; *Sphingomonas* sp. and *Variovorax* sp. promoted the extraction of both metals and plant growth; *Burkholderia* sp. had little effect on phytoextraction	[339]
*Pseudomonas veronii/Sedum alfredii*	Zn	Better plant growth; supplied Fe and P; decreased soil pH	[340]
*Bacillus* sp. E1S2 and *Bacillus pumilus* E2S2/*Sedum plumbizincicola*	Zn and Cd	Production of metal growth promoting mobilizing enzymes and metabolites; improved phytoextraction capacity	[341]
*Pseudomonas* sp. LK9/*Solanum nigrum*	Cu, Cd, and Zn	Organic acids; production of biosurfactants; siderophores	[310]
*Phyllobacterium myrsinacearum/Sedum plumbizincicola*	Pb, Cd, and Zn	Plant growth; improved metal accumulation	[313]

Considering all the advances already made in phytoremediation, together with its undeniable potential to recover or decontaminate soils containing toxic metals, researchers must consider this technique during the selection stage of plants for application in green roofs. With special emphasis on the *Sedum* species, already common in green roofs, it has phytoextraction potential in addition to hyperaccumulation properties. A study that analyzed the quality of runoff water in green roofs compared areas with plants and areas without plants [60]. Green areas containing the species *Portulaca grandiflora* presented high-quality runoff, with low amounts of dissolved solids in the water, low conductivity, and low concentration of metals, especially when compared to the roof without vegetation. These results corroborate the study that also analyzed non-vegetative roofs (n = 4) compared to vegetative roofs (n = 12) for a period of one year [62]. The system without plants presented lower quality in the runoff water compared to the system containing plants. Therefore, plants can increase the quality of wastewater runoff, mainly through their phytoextraction potential. However, this mechanism still needs to be elucidated to increase efficiency and successfully transfer this property to green roofs.

### Integrating Phytoremediation and Biosorption: Synergistic Mechanisms and Design Implications

While Section 4 and Section 5 have independently detailed the pollutant retention capacities of biosorbent-amended substrates and the phytoextraction potential of green roof vegetation, the true performance advantage of next-generation green roofs lies in their dynamic integration. The coupling of biosorption and phytoremediation creates a self-regulating rhizospheric system that overcomes the individual limitations of each process. First, biosorbents act as a protective buffer and slow-release reservoir. By rapidly capturing heavy metals and excess nutrients during initial rainfall pulses, materials such as biochar, seaweed biomass, and crab shell waste reduce the bioavailable metal load in the pore water, thereby mitigating acute phytotoxicity and improving plant survival under stress conditions [210,287]. Concurrently, their high water-holding capacity and gradual nutrient release profiles (particularly from chitin degradation and algal biostimulants) sustain root development during dry periods, directly enhancing the plant’s phytoextraction capacity over seasonal cycles.

Second, vegetation actively modulates biosorbent performance through rhizosphere engineering. Root exudates, including organic acids, amino acids, and phytosiderophores, alter local pH and redox conditions, which can promote the controlled desorption of previously adsorbed metals, making them accessible for plant uptake [331,333]. This rhizosphere-driven mobilization prevents permanent saturation of biosorbent sites and transforms the substrate from a passive sink into a dynamic exchange matrix. Furthermore, the presence of organic amendments stimulates rhizospheric microbial communities that accelerate organic matter decomposition, enhance nutrient mineralization, and participate in metal speciation changes (e.g., Cr(VI) reduction to Cr(III)), collectively amplifying the overall remediation efficiency [341,342,343]. Third, this integration addresses the critical issue of long-term adsorbent exhaustion. In conventional substrate designs, once adsorption sites are saturated, runoff quality deteriorates rapidly. In a coupled biosorption–phytoremediation system, metals are progressively translocated from the substrate to plant shoots, which can be harvested or naturally senesce, effectively exporting contaminants from the roof system. This biological “regeneration” extends the functional lifespan of the biosorbent fraction and reduces the need for costly substrate replacement.

From an engineering perspective, successful integration requires careful matching of amendment kinetics with plant uptake rates. Excessive biosorbent loading (>15% *v*/*v*) may restrict hydraulic conductivity and limit root penetration, while insufficient loading (<5% *v*/*v*) fails to provide adequate buffering against pollutant pulses. Optimal design should prioritize biosorbents with moderate desorption rates under rhizosphere pH conditions, paired with species exhibiting high bioconcentration factors (BCF > 1) and proven tolerance to urban stressors (e.g., *Sedum* spp., *Portulaca grandiflora*). Future pilot-scale studies must quantify the coupled flux of adsorbed versus phytoextracted metals across wet–dry cycles to establish standardized design protocols. Ultimately, framing green roofs as integrated biosorption–phytoremediation systems shifts the paradigm from static substrate engineering to dynamic, biologically driven water quality management. This synergy not only maximizes pollutant removal but also aligns with circular economy principles by leveraging waste-derived materials and vegetation to create self-sustaining urban infrastructure.

## 6. Future Perspectives

Green roofs are a strategy to increase energy savings and manage the problem of rainwater, especially in areas of large urban centers that have highly limited space. Due to the extensive documentation of the advantages of these works on the surface of skyscrapers, several developed countries require their presence in civil construction works to increase sustainability. As a result, several groups of scientists have begun to conduct studies highlighting their positive points. However, after the initial studies, new investigations have begun to address the negative aspects, causing countries to slow down in encouraging the establishment of these green works. The main limitation that needs to be investigated is undoubtedly the quality of the runoff water because several studies confirm a significant source of contaminants that reduce the quality of precipitation water. Despite this, few studies seek to provide solutions that can combat this issue. This scientific article provides strategic methodologies that focus on the selection of plants and the type of substrate to make the presence of green roofs highly sustainable.

To replace the limitations found in frequently used traditional substrates, new mixtures of substrates and plant species are suggested. Among them, three waste-derived biosorbents stand out based on their empirically documented adsorption capacities (mg g^−1^): (i) biochar, with capacities ranging from 5 to 189 mg g^−1^ for nutrients (PO_4_^3−^, NH_4_^+^) and 29–333 mg g^−1^ for heavy metals (Pb, Cd, Cu), depending on feedstock and pyrolysis temperature [248,254,256]; (ii) seaweed biomass (brown, green, red algae), exhibiting 22–360 mg g^−1^ for cationic metals (Pb, Cd, Cu, Zn, Ni) and up to 60 mg g^−1^ for phosphate, with brown algae (e.g., Sargassum, Ascophyllum) generally showing the highest affinities [250,255,258]; and (iii) residual crab shells, achieving 55–870 mg g^−1^ for heavy metals (notably Pb Via microprecipitation) and 109 mg g^−1^ for phosphate, leveraging their chitin and CaCO_3_ content [216,217,219,249]. These quantitative benchmarks provide a rational basis for selecting and dosing biosorbent amendments in green roof substrate design. The selection of these three should focus on materials with adsorption affinity to a wide range of metal contaminants, to make them potent adsorbents and additives in the substrate layer present in the green roof. Long-term research is needed to analyze the compatibility of these substrates with various plant species, thus achieving the maximum potential and benefits that the green roof can provide. All of these investigations should also take into account the cost of the process; therefore, the suggestion of these three groups of substrate materials is focused on materials that are also residual and have practically no cost, such as crab shells. For the emergence of innovative and environmentally friendly materials and their subsequent application in green roofs, extensive studies will be necessary that use new experimental strategies in conjunction with innovative techniques.

Substrate selection is significantly impacted by several factors, including substrate nutrient content, substrate composition mixing ratios, salinity, layer thickness, pH, plant species, thermal conductivity, drought resistance, thermal performance, substrate growth contribution, substrate density, and rainwater retention. It is still unclear to what extent each of these factors interferes with the life cycle of the substrate layer. This knowledge is essential to select suitable substrates for specific green roofs for regions with different climatic conditions. Substrate sustainability must also be supported by a lightweight material with high water retention potential, good thermal performance, adequate and low salinity levels, and good drought resistance, in addition to other related peripheral attributes. Meeting all these factors must also be aligned with the sustainability of climate variations in different countries around the globe. Therefore, researchers in the area need to analyze the climatic specificities that also occur between seasons, supporting decisions in guidelines and legislative standards usually used by developing countries. These guidelines should be specified for regions with dry climates, such as the United Arab Emirates since they present drastic temperature fluctuations affecting evapotranspiration rates. Most substrate studies involve closed environments such as greenhouses or controlled environments and field prototypes. Despite the importance of these studies to understand the impact of each factor on the choice of substrate, they do not fill the gap left by the lack of studies carried out in real conditions, without complete control of climatic variables. Therefore, it is recommended that after carrying out studies with controlled parameters, it is necessary to transfer these preliminary results to the realistic sphere, leading to more sophisticated analyses regarding the use of substrates and the relationships existing in practices inserted in industrial and social contexts.

Regarding the gaps in the experimental field, analyses that encompass thermal performance are needed, especially in long-term investigations. Studies should focus on the interference of the effects of pore distribution and size on soil particles, taking into account the properties of thermal conductivity, water retention, and the light weight of the substrates. These should aim to mitigate the limitations regarding the structural stability present in construction slabs superimposed on green roofs. By filling these gaps, new cultivation media can be selected, which, in addition to being more sustainable, have shown greater adaptation to different climates. These green roofs containing lighter substrates have integrated the architecture of large urban centers, generating a more sustainable and socially pleasant environment. Therefore, these would be the main future scopes for green roof construction projects investigated in the last decade, since the interest of both parties is gradually increasing in the creation of more sustainable engineering structures within urban ecosystems.

## 7. Conclusions

Green roofs have recently been developed as an urban environmentalist solution to several problems that are directly attributed to stormwater runoff and water pollution. This review addresses substrate composition and the synergistic phytoremediation biosorption mechanisms that improve runoff quality. Green roofs have been able to effectively remove a wide range of contaminants, including heavy metals, nutrients, and organic pollutants, by using the natural uptake and degradation potential of plants and the adsorption capacity of biosorbent materials integrated into the substrate. The selection and optimization of substrate materials significantly enhance both pollutant retention and water-holding capacity in green roof systems. Specifically, (i) biochar amendments (5–10% *v*/*v*) achieve 47–86% removal of nutrients (PO_4_^3−^, NO_3_^−^) and 17–75% removal of heavy metals (Pb, Cu, Cd, Zn), with water-holding capacities of 77–79% volumetric retention (~770–790 mg g^−1^); (ii) natural clays (vermiculite, perlite, scoria) exhibit more modest metal removal (15–55% for Pb, Cu, Cd) but provide structural stability and hydraulic conductivity >3600 mm h^−1^, with a water volumetric retention of 25–40% (~250–400 mg g^−1^); and (iii) organic amendments (peat, compost, wood bark) demonstrate 30–86% removal of cationic metals and moderate nutrient buffering, with water-holding capacities of 45–65% volumetric retention (~450–650 mg g^−1^). These quantitative benchmarks provide a rational basis for tailoring substrate formulations to target specific runoff quality objectives while maintaining hydraulic functionality. The result is synergistic; this interplay between phytoremediation and biosorption amplifies the removal of pollutants and enables a portfolio of functions that green roofs foster, including biodiversity, urban cooling, and reuse of water. However, a much deeper understanding is still required for optimal performance, specifically related to the interaction of plants–substrates–pollutants, the longevity of substrates, and maintenance practices. A combination of the different sustainable substrate components with vegetation in green roofs represents a workable and deployable method for mitigating certain urban water quality challenges, furthering sustainable development targets. Future research activities ought to be directed at long-term systems performance, pollutant desorption hazards, and economic feasibility under enhanced substrate design. This could enable the technology to be deployed more widely within city landscaping.

## Figures and Tables

**Table 1:** The most important components present in green roofs according to the published literature.

Type/Details	Vegetative Roof Component	Reference
Geotextile fabric/decreases TSSs (total suspended solids)	Filter layer	[37]
Vegetative roof substrate/5% sphagnum moss, compost and crushed bark; 85% crushed brick	Growth substrate	[47]
Rubber crumbs/energy savings and improved insulation	Drainage element	[48]
Substrate mix/composted organics, crushed brick, coir fiber, and scoria	Growth substrate	[49]
Thermoplastic membranes, liquid-applied membranes, modified bitumen sheets, and single-ply sheet membranes/act as root barriers, prevent leakage and protect the roof	Waterproofing	[34]
Lapillus/cheap option	Drainage element	[50]
Sedum species/used widely in extensive green roofs, succulent, drought tolerant; ground cover	Vegetation	[51,52]
Bioremegree drainage modules/high cost and 2 L/m^2^ water storage capacity	Drainage element	[37]
Norlite (coarse grade expanded shale)/cheap option	Drainage element	[38]
Commercial substrate/shredded peat, crushed lava, clay, and natural calcareous soil	Growth substrate	[53]
*Portulaca* species/ground cover; succulent; used in extensive roofs	Vegetation	[37,54]
Geotextile material/prevents leaching of smaller particles present in the substrate layer	Filter layer	[55]
LECA/cheap drainage element	Drainage element	[56]
Grasses/high biomass and fast growth; reduce water runoff; more root growth	Vegetation	[57]
*Delosperma* species/drought-tolerant; ground cover; succulent	Vegetation	[58,59]
Floradrain FD40/high cost and 4 L m^−2^ water storage capacity	Drainage element	[58]
Vegetative roof substrate/10 and 20% coco-peat; 20% vermiculite and crushed brick; 30% perlite	Growth substrate	[60]

**Table 2:** Comparative summary of key green roof studies evaluating runoff pollutant retention under similar experimental conditions.

System Type and Scale	Substrate/Key Amendment	Vegetation Presence	Target Pollutant(s)	Reported Retention/Removal Trend	Comparative Insight for Runoff Quality	Reference
Pilot-scale extensive	Standard mineral mix ± 7% biochar	*Sedum* spp.	TP, TN, TSS, NO_3_^−^, PO_4_^3−^, turbidity	47–86% reduction in nutrients & suspended solids with biochar amendment	Biochar consistently outperforms unamended mineral media in nutrient binding and particulate filtration	[47]
Real extensive (dry/semi-arid)	Composted organics + crushed brick, coir, scoria	*Myoporum*, *Dianella*, *Lomandra*, *Carpobrotus*	K, PO_4_^3−^, NO_3_^−^, turbidity, pH	High initial K and nutrient leaching; pH stabilization after 6–12 months	Commercial organic-inorganic mixes exhibit early nutrient washout; vegetation moderates’ long-term chemical stability	[62]
Pilot-scale extensive	Substrate + coconut coir vs. control	*Portulaca grandiflora* vs. bare substrate	Al, Cd, Cr, Cu, Fe, Ni, Pb, Zn, TDS	66–88% metal retention with coir; vegetated roofs retained 30–50% more metals than bare substrates	Synergistic coupling of biosorbent + hyperaccumulator plant maximizes metal immobilization vs. substrate-only systems	[60]
Real extensive/intensive (dry climate)	Organic, scoria, brick mixes	Mixed succulents and grasses vs. bare	Ca, NO_3_^−^, Na, NO_2_^−^, NH_4_^+^, PO_4_^3−^	Vegetated systems reduced nutrient leaching by 40–65% compared to bare substrates	Plant uptake is the dominant control on nutrient retention in low-precipitation regimes; substrate alone is insufficient	[62]
Pilot-scale extensive	Commercial peat–mineral mix	*Phedimus takesimensis*, *Sedum*	TP, TN	Moderate retention after initial 3-month leaching phase; seasonal variation significant	Substrate maturation strongly dictates runoff quality; early-stage flushing requires engineered buffering	[63]
Real extensive	Lightweight inorganic aggregates	*Thlaspi*, grasses, *Sedum*	PO_4_^3−^, TP, BOD, pH	High PO_4_^3−^ leaching initially; BOD reduced by 20–35% over 2 years	Aggregate-only substrates lack organic binding sites; integration of waste-derived biosorbents recommended for anion retention	[67]

**Table 3:** Substrate compositions and their compositions used in green roof studies.

Constituent of Substrate	Mix Proportion (%)	Reference
Attapulgite clay	15	[79]
Grape marc compost	15	[79]
Pumice	65	[79]
Zeolite	5	[79]
Peat	10	[19]
Dolomite	5	[19]
Compost yard wastages	3.33	[19]
Composted turkey litter	1.67	[19]
Heat-expanded slate	40	[19]
Sand	40	[19]

**Table 4:** Comparative summary of methodologies for determining plant growth on green roofs.

Methodology	Measurement Principle	Advantages	Limitations	Best Suited for	Representative References
Visual inspection/Photographic analysis	Periodic visual scoring or image-based estimation of coverage, leaf loss, survival	Low cost; non-destructive; suitable for long-term monitoring; scalable to large plots	Subjective; low precision; cannot quantify biomass; requires consistent observer calibration	Extensive roofs with ground-cover species (e.g., *Sedum*, buffalo grass); preliminary screening studies	[88,91]
Three-dimensional pin structure	Vertical pins record hits at canopy peak; coverage = hits/total pins	Quantitative; captures vertical structure; repeatable	Labor-intensive; requires custom apparatus; unsuitable for dense or tall vegetation	Pilot-scale studies with low-growing, horizontally expanding species	[86]
Morphometric measurements (height, diameter, flower count)	Direct manual measurement of plant dimensions and reproductive output	Simple; precise for individual plants; captures multiple growth axes	Time-consuming for large samples; destructive if harvesting required; not scalable to canopy-level metrics	Intensive roofs with discrete individuals; studies linking growth to flowering or biomass	[96]
Simple height measurement (base to apex)	Single vertical measurement per plant	Fast; minimal equipment; easy to standardize	Ignores lateral growth; insensitive to canopy density; poor proxy for biomass	Rapid screening of drought tolerance; comparative studies with uniform morphology	[89]
Binary survival classification (alive/dead)	Visual determination of plant viability at endpoint	Extremely simple; robust for mortality studies; minimal training required	No gradation of stress response; ignores sub-lethal effects; endpoint-only	Drought-resistance screening; long-term survival assessments	[90,91]
Vegetation cover area analysis	Digital image analysis or grid-overlay estimation of % ground covered	Objective; scalable; captures horizontal expansion; compatible with UAV/NDVI	Requires image processing skills; insensitive to vertical growth; affected by lighting conditions	Species with dense, mat-forming growth (e.g., *Portulaca*, *Delosperma*); large-scale monitoring	[93,98]
UAV + NDVI regression modeling	Aerial multispectral imagery + statistical models to predict biomass/coverage	High spatial coverage; non-destructive; enables temporal trend analysis; scalable to city-level	High initial cost; requires technical expertise; model calibration needed per species/climate	Industrial-scale projects; long-term performance monitoring; climate adaptation studies	[98]

**Table 5:** Different pH ranges allowed for crops stored in a greenhouse.

Maximum and Minimum pH	Significance of Ambient pH Range	Greenhouse Crops
5.8 and 5.8	Fe deficiency mitigation and assist blue coloration	Pink hydrangea
5.8 and 5.4	B and Fe deficiency mitigation	Snapdragon
6.8 and 6	Mn and Fe toxicity mitigation	Geranium
6.8 and 6	Mn and Fe toxicity mitigation	Dianthus
5.8 and 5.4	B and Fe deficiency mitigation	Salvia
5.8 and 5.4	Avoid deficiency of B and Fe deficiency and avoid Thielaviopsis	Vinca
5.8 and 5.4	Avoid Thielaviopsis, avoid deficiency of B and Fe	Pansy
6.8 and 6.5	Ca deficiency mitigation and Fe toxicity mitigation	Easter lily
5.6 and 5.2	Contribution to blue coloration and Fe deficiency mitigation	Blue hydrangea
6.8 and 6	Mn and Fe toxicity mitigation	Marigold
6.8 and 6	Mn and Fe toxicity mitigation	Celosia
5.8 and 5.4	Fe and B deficiency mitigation	Petunia
5.8 and 4.5	Fe deficiency mitigation	Azalea

Note: The 13 species were selected from established greenhouse cultivation guidelines [132] to represent (i) a spectrum of pH optima (acidic to neutral), (ii) pH-dependent ornamental traits (e.g., hydrangea flower coloration), and (iii) physiological relevance to green roof vegetation (drought tolerance, shallow roots, low fertility requirements). The pH ranges listed reflect species-specific optima for ornamental greenhouse crops, particularly those with pH-dependent traits (e.g., hydrangea flower coloration). These values are derived from controlled cultivation studies [132] and may differ from the generalized FLL-recommended substrate pH range of 6.0–8.5 for green roofs, which prioritizes broad compatibility, structural stability, and long-term resilience under variable urban conditions.

**Table 6:** Inorganic constituents present in green roofs and their respective sorption capacities.

Inorganic Constituent/Metals	K_d_ (L g^−1^)	Adsorption Capacity (mg g^−1^)	Reference
Vermiculite/Pb	35.29	49	[197]
Crushed brick/Cl	1.90	13	[198]
Scoria/Cd	0.07	2.4	[194]
Perlite/Ni	1.85	3.3	[196]
Sand/Cl	1.66	2.2	[198]
Vermiculite/Cu	11.64	12.6	[197]
Crushed brick/PO_4_	1.00	18.2	[198]
Scoria/Zn	0.16	6.2	[199]
Vermiculite/Cd	4.41	11.1	[197]
Scoria/Cu	0.08	1.7	[194]
Sand/PO_4_	0.97	3.3	[198]
Sand/NO_3_	0.94	5.8	[198]
Pumice/Cr(III)	61.71	1.6	[195]
Scoria/Zn	0.10	1.5	[194]
Crushed brick/NO_3_	1.95	14	[198]
Sand/Surfactant	0.958	2.16	[198]
Vermiculite/Ni	3.29	6.75	[197]
Perlite/Cd	0.26	2.8	[196]
Perlite/Pb	7.74	27	[196]
Vermiculite/Pb	4.41	5	[197]
Crushed brick/Surfactant	1.35	2.5	[198]
Scoria/Pb	0.15	6.9	[194]
Sand/Cu(II)	0.25	2.04	[200]
Perlite/Cu	3.11	5.6	[196]
Scoria/As(III)	0.00	1.6	[194]
Pumice/Cu	33.79	3.5	[195]

Notes: The distribution coefficient (K_d_) was estimated from Langmuir isotherm parameters for the linear region using K_d_ = Q_max_ × K_L_, where Q_max_ is the maximum adsorption capacity and K_L_ is the Langmuir affinity constant.

**Table 7:** Organic constituents present in green roof substrates and their respective adsorption capacities.

Organic Constituent/Metals	K_d_ (L g^−1^)	Adsorption Capacity (mg g^−1^)	Reference
Green waste compost/Cu	1.39	30	[206]
Mulch (*Madhuca longifolia*)/Pb	0.31	17.2	[207]
Peat/Pb	4.32	48	[206]
Coir pith/Cr	0.02	11.6	[204]
Mulch (Hardwood)/Pb	19.17	72.5	[202]
Wood bark/Zn	0.25	11	[206]
Green waste compost/Zn	1.37	14	[206]
Coir pith/Ni	0.04	16	[204]
Mulch (Hardwood)/Cu	2.64	23	[202]
Bark compost/Cu	0.99	4.7	[208]
Mulch (*Polyalthia longifolia*)/Pb	0.34	4.5	[207]
Bark compost/Zn	1.41	2.6	[208]
Coir pith/Co	0.01	13	[204]
Peat/Zn	0.17	4.2	[206]
Mulch (Hardwood)/Zn	5.03	12.2	[202]
Green waste compost/Cu	0.66	30	[206]
Bark compost/Ni	0.56	0.7	[208]
Coir pith/Cr (VI)	1.98	165	[205]
Peat/Cu	0.40	12	[206]
Green waste compost/Pb	8.91	86	[206]
Wood bark/Cu	0.58	17	[206]
Bark compost/Pb	0.97	7.7	[208]

Notes: The distribution coefficient (K_d_) was estimated from Langmuir isotherm parameters for the linear region using K_d_ = Q_max_ × K_L_, where Q_max_ is the maximum adsorption capacity and K_L_ is the Langmuir affinity constant.

**Table 9:** List of plant species with phytoremediation potential and possible application in green roofs, including bioconcentration (BCF) and translocation (TF) factors where reported.

Metals/Plant Species	BCF/TF	Metal Accumulation (mg kg^−1^)	Reference
Cd/*Solanum nigrum*	-/3.27 ^†^	117.2 (leaf); 77.0 (stem); 35.9 (root)	[310]
Pb/*Melastoma malabathricum*	1.8 ^‡^/-	2390 (overall)	[311]
Cd/*Melastoma malabathricum*	2.1 ^‡^/-	426 (overall)	[311]
Zn/*Solanum nigrum*	-/0.51 ^†^	85.5 (leaf); 95.4 (stem); 167.9 (root)	[310]
Pb/*Helichrysum italicum*	-/1.4	484 (shoot); 346 (root)	[312]
Pb/*Sedum plumbizincicola*	3.2 ^‡^/1.02	101 (shoot); 99 (root)	[313]
*Cd/Sedum alfredii*	8.5 ^‡^/6.74	137 (root); 923 (shoot)	[314]
Zn/*Helichrysum italicum*	-/1.82	1176 (shoot); 646 (root)	[312]
Cu/*Melastoma malabathricum*	1.5 ^‡^/-	1820 (overall)	[311]
Cu/*Solanum nigrum*	-/0.50 ^†^	32.2 (leaf); 12.3 (stem); 64 (root)	[310]
Pb/*Portulaca grandiflora*	-	9.77 (overall)	[315]
Cd/*Pennisetum purpureum*	-	1.3–7.05 (shoot)	[316]
Zn/*Ficus macrocarpa*	0.9 ^‡^/-	561 (overall)	[311]
Cd/*Sedum plumbizincicola*	2.8 ^‡^/2.66	93 (shoot); 35 (root)	[313]
Cr (VI)/*Portulaca oleracea*	-/0.3	1400 (stems); 4600 (roots)	[317]
Pb/*Ficus macrocarpa*	1.1 ^‡^/-	1050 (overall)	[311]
Zn/*Melastoma malabathricum*	2.3 ^‡^/-	1380 (overall)	[311]
Cd/*Ficus macrocarpa*	1.7 ^‡^/-	419 (overall)	[311]
Zn/*Sedum plumbizincicola*	4.1 ^‡^/1.21	1072 (shoot); 889 (root)	[313]
Cu/*Ficus macrocarpa*	1.4 ^‡^/-	1260 (overall)	[311]

Note: BCF = C_root_/Csubstrate; TF = C_shoot_/C_root_. Values >1 indicate hyperaccumulator potential; ^†^ Calculated from reported tissue concentrations: TF = (leaf + stem)/2/root. ^‡^ Approximate BCF estimated from source study substrate concentrations; actual values may vary with experimental conditions. “-” indicates data not reported in source study.

## Data Availability

No new data were created or analyzed in this study.

## Abbreviations

The following abbreviations are used in this manuscript:

Abbreviation	Meaning
TSS	Total Suspended Solid
COD	Chemical Oxygen Demand
PO_4_	Phosphate
NO_3_	Nitrate
TP	Total Phosphorus
TN	Total Nitrogen
TDS	Total Dissolved Solid
TOC	Total Organic Carbon
BOD	Biochemical Oxygen Demand
BET	Brunauer–Emmett–Teller
ASTM	American Society for Testing and Materials
FLL	Forschungsgesellschaft Landschaftsentwicklung Landschaftsbau
NDVI	Normalized Difference Vegetation Index
NPK	Nitrogen, Phosphorus, Potassium
C_root_, C_shoot_, C_substrate_	Metal concentration in roots, shoots, and substrate
